# Phosphorylation of a Central Clock Transcription Factor Is Required for Thermal but Not Photic Entrainment

**DOI:** 10.1371/journal.pgen.1004545

**Published:** 2014-08-14

**Authors:** Euna Lee, Eun Hee Jeong, Hyun-Jeong Jeong, Evrim Yildirim, Jens T. Vanselow, Fanny Ng, Yixiao Liu, Guruswamy Mahesh, Achim Kramer, Paul E. Hardin, Isaac Edery, Eun Young Kim

**Affiliations:** 1 Neuroscience Graduate Program, Department of Biomedical Sciences, Ajou University School of Medicine, Suwon, Kyunggi-do, Republic of Korea; 2 Department of Brain Science, Ajou University School of Medicine, Suwon, Kyunggi-do, Republic of Korea; 3 Department of Molecular Biology and Biochemistry, Rutgers University, Center for Advanced Biotechnology and Medicine, Piscataway, New Jersey, United States of America; 4 Laboratory of Chronobiology, Charité–Universitätsmedizin, Berlin, Germany; 5 Texas A&M University Department of Biology and Center for Biological Clocks Research, College Station, Texas, United States of America; Florida State University, United States of America

## Abstract

Transcriptional/translational feedback loops drive daily cycles of expression in clock genes and clock-controlled genes, which ultimately underlie many of the overt circadian rhythms manifested by organisms. Moreover, phosphorylation of clock proteins plays crucial roles in the temporal regulation of clock protein activity, stability and subcellular localization. dCLOCK (dCLK), the master transcription factor driving cyclical gene expression and the rate-limiting component in the *Drosophila* circadian clock, undergoes daily changes in phosphorylation. However, the physiological role of dCLK phosphorylation is not clear. Using a *Drosophila* tissue culture system, we identified multiple phosphorylation sites on dCLK. Expression of a mutated version of dCLK where all the mapped phospho-sites were switched to alanine (dCLK-15A) rescues the arrythmicity of *Clk* ^out^ flies, yet with an approximately 1.5 hr shorter period. The dCLK-15A protein attains substantially higher levels in flies compared to the control situation, and also appears to have enhanced transcriptional activity, consistent with the observed higher peak values and amplitudes in the mRNA rhythms of several core clock genes. Surprisingly, the clock-controlled daily activity rhythm in dCLK-15A expressing flies does not synchronize properly to daily temperature cycles, although there is no defect in aligning to light/dark cycles. Our findings suggest a novel role for clock protein phosphorylation in governing the relative strengths of entraining modalities by adjusting the dynamics of circadian gene expression.

## Author Summary

Circadian clocks are synchronized to local time by daily cycles in light-dark and temperature. Although light is generally thought to be the most dominant entraining cue in nature, daily cycles in temperature are sufficient to synchronize clocks in a large range of organisms. In *Drosophila*, dCLOCK is a master circadian transcription factor that drives cyclical gene expression and is likely the rate-limiting component in the transcriptional/translational feedback loops that underlie the timekeeping mechanism. dCLOCK undergoes temporal changes in phosphorylation throughout a day, which is also observed for mammalian CLOCK. However, the role of CLOCK phosphorylation at the organismal level is still unclear. Using mass-spectrometry, we identified more than a dozen phosphorylation sites on dCLOCK. Blocking global phosphorylation of dCLOCK by mutating phospho-acceptor sites to alanine increases its abundance and transcriptional activity, leading to higher peak values and amplitudes in the mRNA rhythms of core clock genes, which likely explains the accelerated clock speed. Surprisingly, the clock-controlled daily activity rhythm fails to maintain synchrony with daily temperature cycles, although there is no observable defect in aligning to light/dark cycles. Our findings suggest a novel role for clock protein phosphorylation in governing the effective strengths of entraining modalities by adjusting clock amplitude.

## Introduction

A large variety of life forms manifest circadian (≅24 hr) rhythms in behavior and physiology that are driven by endogenous cellular clocks or pacemakers [1], [2]. Perhaps the most biologically relevant property of circadian clocks is that they can be synchronized (entrained) to local time by external time cues, a feature that endows organisms with the ability to anticipate environmental changes and hence perform activities at optimal times during the day. The main environmental synchronizing agents of circadian clocks in nature are the daily cycles in light/dark and ambient temperature. In general, photic cues are the most potent synchronizing agent for organisms, whereas thermal entrainment is less powerful [3], [4]. Work in the last 20 years using a variety of model organisms has revealed that the molecular logic underlying circadian clock mechanisms is highly conserved [2]. Circadian clocks are based on intracellular mechanisms that involve a core transcriptional translational feedback loop (TTFL) composed of central clock proteins that drive daily oscillations in their own gene expression as well as downstream clock-controlled genes (ccgs). Daily oscillations in the transcript levels of ccgs ultimately drive many of the rhythmic behaviors and physiologies manifested by organisms.

The rate-limiting component of the main TTFL in *Drosophila* is the basic-helix-loop-helix (bHLH) PAS domain containing transcription factor termed dCLOCK (*Drosophila* CLOCK; dCLK) [5], which forms a heterodimer with CYCLE (CYC), another bHLH-PAS containing clock transcription factor [6]. The dCLK-CYC heterodimer binds to E box DNA elements inducing the expression of the clock genes *period* (*per*) and *timeless* (*tim*), in addition to other clock genes and ccgs. Subsequently, the PER and TIM proteins interact in the cytoplasm and after a time-delay translocate to the nucleus where they function with other factors to inhibit the transcriptional activity of dCLK-CYC. Eventually, the levels of PER and TIM decline in the nucleus, facilitating another round of dCLK-CYC-mediated transcription. In a “secondary” stabilizing TTFL, the dCLK-CYC heterodimer induces the expression of *PAR domain protein 1ε* (*pdp1ε*) and *vrille* (*vri*), whose protein products (i.e., PDP1ε and VRI) in turn activate and repress the expression of *dClk*, respectively, leading to daily cycles in *dClk* mRNA levels [7], [8]. Mammalian clocks also use a CLOCK-based transcription factor in their main TTFL, which involves a heterodimer comprised of mCLOCK (mammalian CLOCK; mCLK) and BMAL1 (homolog of CYC) that governs rhythmic expression of the negative regulators *Per*1-3, in addition to other clock genes and ccgs [9].

Although TTFLs constitute a major molecular framework for the oscillatory behavior of cellular clocks, posttranslational modifications of clock proteins are central to maintain proper timekeeping functions by regulating clock protein stability, sub-cellular localization and activity [10]–[14]. A well-studied example of clock protein phosphorylation is the progressive phosphorylation of PER, which has a critical role in setting the pace of the clock and controlling temporal changes in dCLK-CYC-mediated transcription by regulating PER stability, timing of nuclear entry and how long it persists in the nucleus [15]–[22]. Newly synthesized PER is present as non-to-hypo-phosphorylated isoforms in the late day/early night and undergoes progressive increases in the extent of phosphorylation, culminating in the appearance of mostly or exclusively hyper-phosphorylated isoforms in the late night/early day that are recognized for rapid degradation by the 26S proteasome [10]. Numerous PER-relevant kinases have been identified, with DOUBLETIME [DBT; homolog of vertebrate casein kinase Iδ/ε (CKIδ/ε)] [21], [23] operating as the major kinase regulating temporal changes in the stability of PER. Other kinases include SHAGGY [SGG; homolog of vertebrate glycogen synthase kinase 3β (GSK3β)] [24], casein kinase 2 (CK2) [25], [26] and NEMO [15], [27].

dCLK also undergoes circadian changes in phosphorylation state, but in a manner different from that of PER [28], [29]. dCLK is present in a mostly intermediate phosphorylated state throughout the day, converting to largely hyper-phosphorylated isoforms in the late night/early day. DBT stably interacts with PER throughout most of its daily life-cycle and this association likely facilitates the ability of DBT to regulate dCLK [28]–[31]. Although the role(s) of dCLK phosphorylation is not clear it appears that hyper-phosphorylated isoforms have decreased stability and possibly reduced transcriptional activity [28]–[30]. In addition to DBT, several kinases such as protein kinase A (PKA), CaMKII, MAPK, and NMO have been implicated in regulating the activity and/or levels of dCLK [27], [32]. More recently CK2 was reported to act directly on dCLK, stabilizing it while reducing its activity [33]. The mammalian CLOCK protein also manifests circadian oscillations in phosphorylation *in vivo* [34], [35], which is triggered by hetero-dimeric complex formation with BMAL1 [34], [35]. Mass spectrometric analysis of purified mCLK from the mouse liver identified Ser38, Ser42, and Ser427 as sites phosphorylated *in vivo* [36]. Ser38 and Ser42 are located in the bHLH region and phosphorylation of those residues down-regulates transcriptional activity of mCLK via decreasing binding activity to E box element [36]. Phosphorylation of Ser427 is reported as being dependent on GSK-3β activity and relevant for degradation of mCLK [37]. PKG and PKC have been implicated as mCLK kinases regulating phase resetting [38], [39]. Despite these advances using several animal model systems, it is still unclear how CLOCK phosphorylation impacts the function of circadian timing systems at the organismal level.

In this study, we used a simplified *Drosophila* S2 cell culture system in combination with mass spectrometry to map phosphorylation sites on dCLK. Our results indicate that dCLK is highly phosphorylated (at least 14 phospho-sites). In S2 cells, mutated versions of dCLK where all the mapped Ser/Thr sites were switched to Ala (herein referred to as dCLK-15A) manifested increased E box dependent transcriptional activity without affecting interactions with other core clock partners such as CYC and PER. In flies, dCLK-15A protein is exclusively hypo-phosphorylated suggesting that we identified, at the very least, a major portion of the total phosphorylation sites found on dCLK in flies. Expression of dCLK-15A rescues the arrhythmicity of *Clk* ^out^ flies yet with an approximately 1.5 hr shorter period. Consistent with a role in regulating protein stability, the levels of dCLK15A are substantially higher compared to the control situation, which along with increases in transcriptional activity likely explains the faster pace of the clock. The daily peak levels in *per*/*tim* mRNA and protein reached higher values in dCLK-15A expressing flies, further supporting the notion that dCLK levels are normally rate-limiting in the clock mechanism. Surprisingly, the clock-controlled daily activity rhythm in dCLK-15A mutant flies fail to maintain synchrony with daily temperature cycles, although there is no defect in aligning to light/dark cycles. Together, our findings indicate that in animal systems, the post-translational modification of a master circadian transcription factor plays a critical role in setting the pace of the clock and regulating circadian entrainment.

## Results

### Identification of dCLK phosphorylation sites in cultured *Drosophila* cells

**Figure 1:**
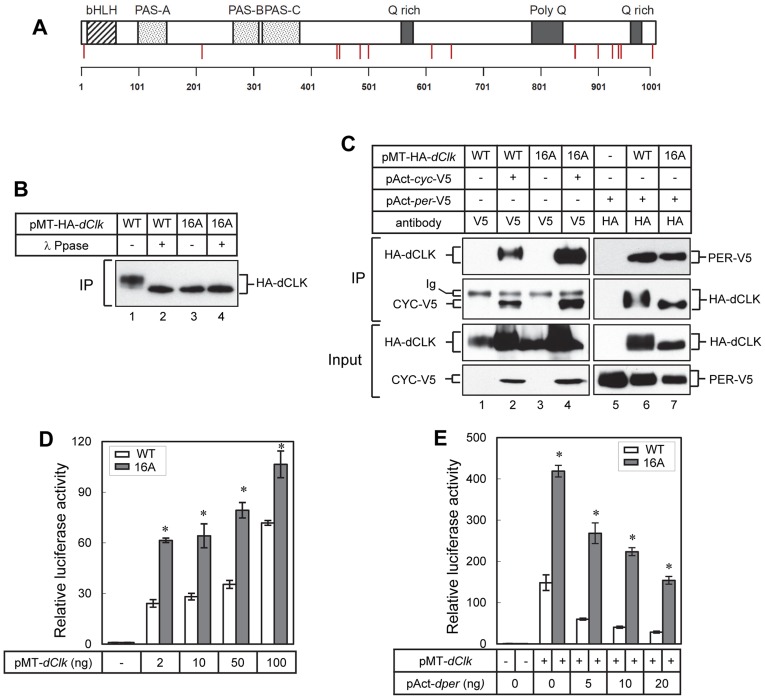
Blocking phosphorylation at multiple phospho-sites on dCLK prevents global phosphorylation but does not impair several key clock-relevant activities. (A) Schematic diagram of dCLK protein. Phosphorylation sites on dCLK identified in this study are indicated as red vertical lines. Horizontal line at bottom indicates relative positions of amino acid residues. (B, C) S2 cells were transiently transfected with 500 ng of pMT-HA-*dClk* (WT) or pMT-HA-*dClk*-16A (16A), either singly or in combination with 500 ng of pAct-*cyc*-V5 or pAct-*per*-V5 as indicated. Expression of dCLK was induced 24 hr after transfection by adding 500 µM CuSO_4_ to the medium. Cells were harvested 24 hr after induction, and protein extracts were first subjected to immunoprecipitation using anti-HA (12CA5) antibody (B), the anti-epitope tag antibodies (V5 or HA) as indicated on top of the blots (C). Immune complexes were directly analyzed by immunoblotting (C) or further incubated in the absence (−) or presence (+) of λ phosphatase followed by immunoblotting (B). (D, E) S2 cells were transiently co-transfected either singly with pMT-*dClk*-V5 (WT) and pMT-*dClk*-16A-V5 (16A) (D), or in combination with increasing amount of pAct-*per* (E). Shown are the average values from three independent experiments for relative E box dependent luciferase activity in the absence (−) or presence (+) of pMT-*dClk*-V5. *p<0.05; error bars denote SEM.

As an initial attempt to better understand the role(s) of dCLK phosphorylation we sought to map phosphorylation sites using recombinant protein production in cultured *Drosophila* S2 cells. This simplified cell culture system was successfully used to identify physiologically relevant phosphorylation sites on *Drosophila* PER [15], [16], [18], [40]. Prior work showed that production of recombinant dCLK in S2 cells leads to significant shifts in electrophoretic mobility that are due to phosphorylation [29]. Thus, we established S2 cell lines stably expressing HA-*dClk*-V5 under the inducible metallothionein promoter (pMT). Total cell lysates were subjected to immunoprecipitation with anti-V5 antibody, followed by multi-protease digestion, titansphere nanocolumn phosphopeptide enrichment, and tandem mass spectrometry, as previously described [16], [41]. We identified 14 phosphorylation sites on dCLK, all of which are at Ser residues, with the possible exception of Tyr607 (Table 1). Many of the identified phosphorylation sites are in the C-terminal half of dCLK, which contains several Q-rich regions that might function in transcriptional activation (Figure 1A). Phosphorylation was also detected at sites close to the N- and C-terminus of the dCLK protein. Interestingly, no phosphorylation sites were found in any of the known functional domains of dCLK; e.g. bHLH, PAS domains and Q-rich regions (Figure 1A and Table 1).

**Table 1:** Identification of phosphorylation sites on dCLK produced in *Drosophila* S2 cells.

dCLK^a^
S5^b^
[S209,S210,S211]^c^
S444
S450
S487
S504
[Y607,S611]^c^
S645
S859
S902
S924
S934
S938
S1018

a Stable cell line expressing *dClk* under the control of pMT-inducible promoter.

b Amino acids are numbered according to sequence of dCLK (amino acids 1–1027), GenBank accession number NP_001014576.

c Only one site in the bracket is phosphorylated.

In preliminary studies we individually mutated each of the identified phosphorylated Ser residues to Ala residues but did not see major effects on dCLK electrophoretic mobility, except for the S859A mutant version of dCLK, which manifested slightly faster electrophoretic mobility. (Figure S1A and B). The transcriptional activities of most single site mutants were somewhat increased (≤2 fold), except for the S924A mutant version of dCLK, which manifested a slight but reproducible decrease (Figure S1C). Overall, our initial studies in S2 cells were not able to identify whether certain individual phospho-sites are particularly significant in regulating dCLK metabolism or activity. While ongoing work is aimed at better understanding the roles of individual phospho-sites, in this study we focused on more global aspects of dCLK phosphorylation by generating a mutant version wherein all the Ser phospho-acceptor sites identified by mass spectrometry were switched to Ala. Since the mass spectrometry data did not unambiguously identify which Ser among amino acids 209–211 is phosphorylated, we switched all 3 Ser to Ala. In addition, although Tyr 607 or Ser 611 is phosphorylated, to focus on Ser phosphorylation, we only mutated Ser 611 to an Ala. By using site-directed mutagenesis, we serially mutated the aforementioned 16 Ser to Ala (dCLK-16A). The electrophoretic mobility of dCLK-16A is indistinguishable from that of λ-phosphatase treated wild-type dCLK and was not altered by λ-phosphatase treatment (Figure 1B), indicating that we mapped most, if not all, the sites on dCLK phosphorylated by endogenous kinases in S2 cells.

dCLK-16A interacts with either CYC or PER proteins to a similar extent as that observed for the wild-type version (dCLK-WT), demonstrating that dCLK-16A is not grossly misfolded (Figure 1C). In addition, our findings suggest that the phosphorylated state of dCLK is not a major signal regulating interactions with core clock partners. Consistent with prior work, we observed increases in non/hypo-phosphorylated isoforms of dCLK when dPER is co-expressed (Figure 1C, compare lane 1 and 6) [31]. With regards to transcriptional activity, dCLK-16A is more potent compared to dCLK-WT in stimulating E-box dependent transcription (Figure 1D), while still maintaining its sensitivity to inhibition by dPER (Figure 1E). Earlier findings showed that hyper-phosphorylated dCLK is less stable and that DBT might contribute to this instability, although the exact role of DBT is not clear [28]–[30]. We compared the stabilities of dCLK-16A and dCLK-WT under a variety of conditions, including overexpressing DBT, but did not detect a significant difference (Figure S2A and B), suggesting we did not map one or more phosphorylation sites critical for regulating dCLK stability and/or the pathway for dCLK degradation in S2 cells is not identical to that in flies (see below). Taken together, the results obtained using well-established S2 cell based assays indicate that dCLK-16A retains key clock-relevant biochemical functions and suggest that global phosphorylation of dCLK reduces its transactivation potential.

### Flies expressing dCLK-15A display behavioral rhythms with short periods

To investigate whether the dCLK phosphorylation sites we identified play a physiological role in the *Drosophila* circadian timing system, we first evaluated the ability of a novel wildtype *dClk* transgene to rescue behavioral rhythms in the arrhythmic *Clk* ^out^ genetic background (herein, termed as p{*dClk*-WT};*Clk* ^out^). *Clk* ^out^ is a newly described arrhythmic null mutant that does not produce dCLK protein (Mahesh et al., submitted). The transgene was constructed with a 13.9 kb genomic fragment that contains the *dClk* gene, which we modified by introducing a V5 epitope tag at the C-terminus of the *dClk* open reading frame for enhanced protein surveillance. Flies were exposed to standard entraining conditions of 12 hr light∶12 hr dark cycles [LD; where zeitgeber time 0 (ZT0) is defined as lights-on] at 25°C, followed by several days in constant dark conditions (DD) to measure free-running behavioral periods. In the behavioral analysis, p{*dClk*-WT};*Clk* ^out^ flies manifested robust locomotor activity rhythms with normal ∼24 hr periods (Table 2, Mahesh et al., submitted), indicating that the circadian clock system functions properly in these flies.

**Table 2:** Behavioral analysis of p{*dClk*-15A}; *Clk* ^out^ flies following light/dark entrainment.^a^

Genotype	Temp (°C)	Number^b^	Tau ± S.E.M. (h)	Rhythmicity (%)^c^	Power^d^
p{*dClk*-WT}, A;*Clk* ^out^	18	30	24.0±0.08	66.7	39.8
p{*dClk*-15A}, 2M; *Clk* ^out^	18	28	22.2±0.09	71.4	47.8
p{*dClk*-15A}, 6M; *Clk* ^out^	18	42	22.1±0.12	33.3	43.0
*Clk* ^out^	25	19	AR	AR	AR
p{*dClk*-WT}, A;*Clk* ^out^	25	63	24.0±0.08	87.3	114.7
p{*dClk*-15A}, 2M; *Clk* ^out^	25	39	22.3±0.07	61.5	67.4
p{*dClk*-15A}, 6M; *Clk* ^out^	25	42	22.7±0.08	73.8	67.2
p{*dClk*-WT}, A;+/+	25	29	23.0±0.07	48.3	62.8
p{*dClk*-WT}, A;*Clk* ^out^	29	30	24.1±0.05	83.3	113.5
p{*dClk*-15A}, 2M; *Clk* ^out^	29	24	22.5±0.5	12.5	86.4
p{*dClk*-15A}, 6M; *Clk* ^out^	29	14	22.3±0.12	35.7	51

a Flies were kept at indicated temperatures (18°C, 25°C, 29°C) and exposed to 4 days of 12∶12 LD followed by 7 days of DD.

b Total number of flies that survived until the end of the testing period.

c Percentage of flies with activity rhythms having a power value of ≥10 and a width value of ≥2.

d Measure of the strength or amplitude of the rhythm.

Next, we sought to generate transgenic flies harboring a dCLK-16A version of the *dClk* rescue transgene. However, because of technical difficulties in generating a version that also included replacing Ser5 with Ala, we made a *dClk* version wherein the other 15 Ser residues were switched to Ala, termed *dClk*-15A. In S2 cells, dCLK-15A behaves similar to dCLK-16A, including no observable effect of phosphatase treatment on electrophoretic mobility and enhanced E-box dependent transcriptional activity (Figure S3). Although phosphorylation of Ser5 might affect dCLK function in a manner that is not revealed in the S2 cell based assays we used, the CLK-15A protein contains the majority of phosphorylation sites and should address if global phosphorylation of dCLK plays an important role in the circadian timing system. Two independent lines of transgenic flies harboring the *dClk*-15A transgene were obtained and circadian behavior was monitored in the *Clk* ^out^ genetic background (referred to as p{*dClk*-15A}, 2M; *Clk* ^out^ and p{*dClk*-15A}, 6M; *Clk* ^out^). In sharp contrast to flies harboring the control version of *dClk*, the two independent lines of p{*dClk*-15A};*Clk* ^out^ flies manifested generally weaker behavioral rhythms that are approximately 1.5 hr shorter compared to their wild-type counterparts (Table 2).

**Figure 2:**
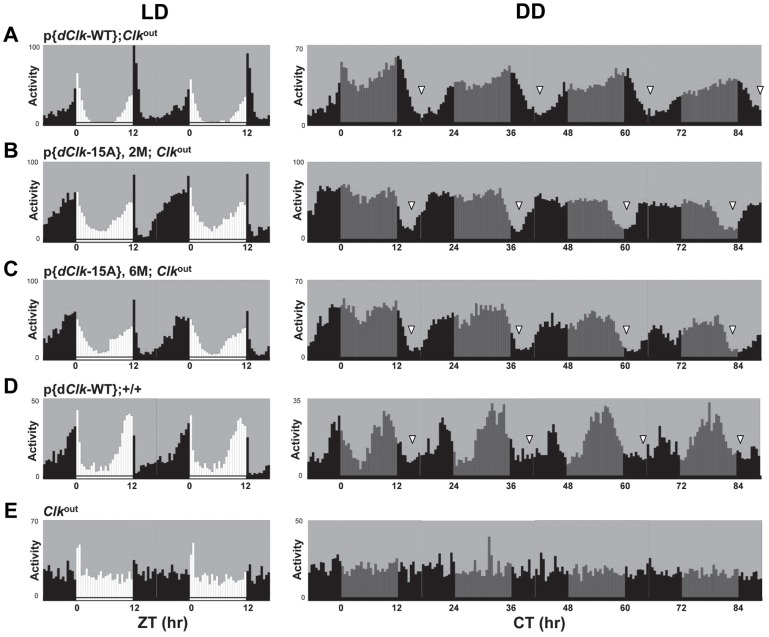
(A–E) The p{*dClk*-15A};*Clk* ^out^ flies manifest short period behavioral rhythms. Each panel represents the average activity of male flies for a given genotype during the third and fourth day of 12 hr light∶12 hr dark entrainment (LD) followed by 4 days of constant darkness (DD). White vertical bars represent locomotor activity during light phase and black vertical bars represent locomotor activity during dark phase in LD. Gray vertical bars represent locomotor activity during the subjective light phase in DD. White horizontal bars and black horizontal bars below each panel indicate 12 hr periods of lights-on and lights-off, respectively. Arrowheads indicate the times in a daily cycle when trough levels of activity were attained following the evening bout of activity. Standard error of the mean is indicated as dots above each bars.

Under standard conditions of LD at 25°C, *D. melanogaster* exhibits a bimodal distribution of activity with a “morning” and “evening” bout of activity centered around ZT0 and ZT12, respectively. Although p{*dClk*-15A};*Clk* ^out^ flies manifest the typical bimodal distribution of locomotor activity, the onset of both the morning and evening bouts of activity were earlier (Figure 2, compare panels B and C to A), consistent with the shorter free-running periods. The *Clk* ^out^ flies only showed a “startle” response to the lights-on transition but no rhythmic behavior (Figure 2E). In constant dark conditions, the downswing in evening activity is clearly earlier in p{*dClk*-15A};*Clk* ^out^ flies, in agreement with the shorter free-running period (Table 2, Figure 2 and S4). We also examined the locomotor behavior of flies harboring the *dClk*-WT transgene in a wild-type genetic background, resulting in flies with four copies of the *dClk* gene (herein referred to as p{*dClk*-WT};+/+). The circadian period was shortened to approximately 23 hr (Table 2 and Figure 2D), which is well correlated with previous reports demonstrating that increasing the copy number of *dClk* shortens the circadian period of behavioral rhythms [42], [43].

A hallmark property of circadian rhythms is that the period length is very constant over a wide range of physiologically relevant temperatures, termed temperature compensation [44]. To investigate whether phosphorylation of dCLK might have a role in temperature compensation, we analyzed behavioral rhythms at three standard temperatures (i.e., 18°, 25° and 29°C). Although we noted a decrease in rhythmicity for *dClk*-15A;*Clk* ^out^ flies at 29°C, the periods were quite similar over the temperature range tested (Table 2), suggesting that global phosphorylation of dCLK does not play a major role in temperature compensation.

### dCLK-15A is exclusively hypo-phosphorylated and very abundant in flies

**Figure 3:**
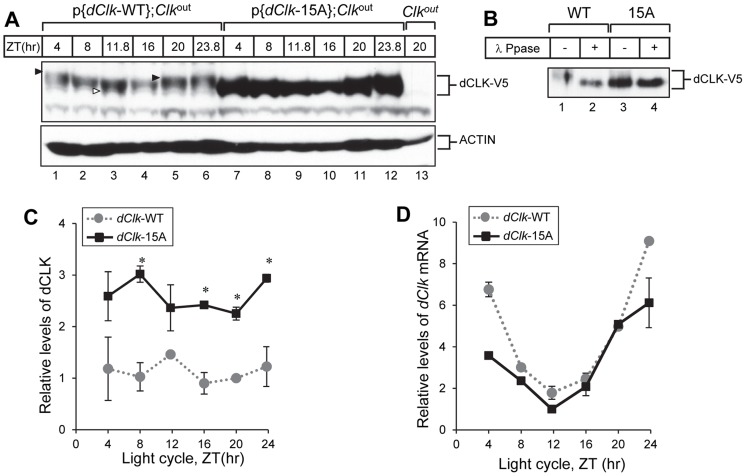
The levels of dCLK are substantially higher and hypo-phosphorylated at all times of the day in p{*dClk*-15A};*Clk* ^out^ flies. (A–C) Adult flies of the indicated genotype were collected at different times of day (ZT), head extracts prepared and directly analyzed for immunoblotting (A) or processed for immunoprecipitation with anti-V5 Ab (B). β-Actin (ACTIN) served as a loading control. Immune complexes were further incubated in the absence (−) or presence (+) of λ phosphatase and immunoblotted with anti-V5 antibody, as indicated (B). Filled arrowheads denote hyper-phosphorylated isoforms of dCLK and open arrowhead denotes hypo-phosphorylated isoforms of dCLK (A). (C) Relative levels of dCLK were determined by measuring staining intensities using image J software. Shown are the average values from three independent experiments. (D) Total RNA was extracted from fly heads, and quantitative real-time RT-PCR was performed to measure the relative levels of *dClk* transcripts. Shown are the average values from three independent experiments using p{*dClk*-15A}, 6M;*Clk* ^out^ flies. Error bars denote SEM.

We examined the temporal profiles of dCLK protein by analyzing head extracts prepared from p{*dClk*-WT};*Clk* ^out^ and p{d*Clk*-15A};*Clk* ^out^ flies in LD conditions (Figure 3A and C). dCLK-WT protein undergoes daily changes in phosphorylation that are consistent with earlier results probing endogenously produced dCLK; namely, hypo- to medium- phosphorylated isoforms present during the mid-day/early night (e.g., ZT 8 to ZT 16) and mostly hyper-phosphorylated isoforms present in the late night/early day (e.g., ZT20 to ZT4) (Figure 3A) [28], [29]. However, the mobility of dCLK-15A was similar throughout a daily cycle (Figure 3A), and co-migrated with λ phosphatase treated dCLK-WT (Figure 3B). Thus, similar to results in S2 cells, dCLK-15A exhibits little to no phosphorylation *in vivo*, suggesting that the phospho-sites we identified by mass spectrometry comprise, at the very least, a major portion of the total phosphorylation sites found on dCLK in flies (it is also possible that one or more of the phospho-sites we mutated are required for phosphorylation at other sites, but this would still result in a mainly hypo-phosphorylated dCLK protein). Intriguingly, the levels of dCLK-15A were substantially higher compared to dCLK-WT throughout a daily cycle. Quantification of immunoblots indicated that the average daily levels of dCLK-15A are about 2.5 times more than those of dCLK-WT (Figure 3C).

To examine whether the high levels of dCLK-15A proteins results from elevated mRNA abundance, we measured *dClk* transcript levels. As reported previously, although the overall daily abundance of dCLK-WT protein is essentially constant throughout a daily cycle, *dClk*-WT mRNA levels oscillate with peak amounts attained during the late night-to-early day and reaching trough values around ZT12 [45], [46]. The daily oscillation in *dClk*-15A mRNA abundance is similar to the wild-type situation and even seemed to have lower peak levels (Figure 3D). These results indicate that in general global phosphorylation of dCLK decreases its stability *in vivo*, consistent with prior findings using S2 cells [28], [29].

**Figure 4:**
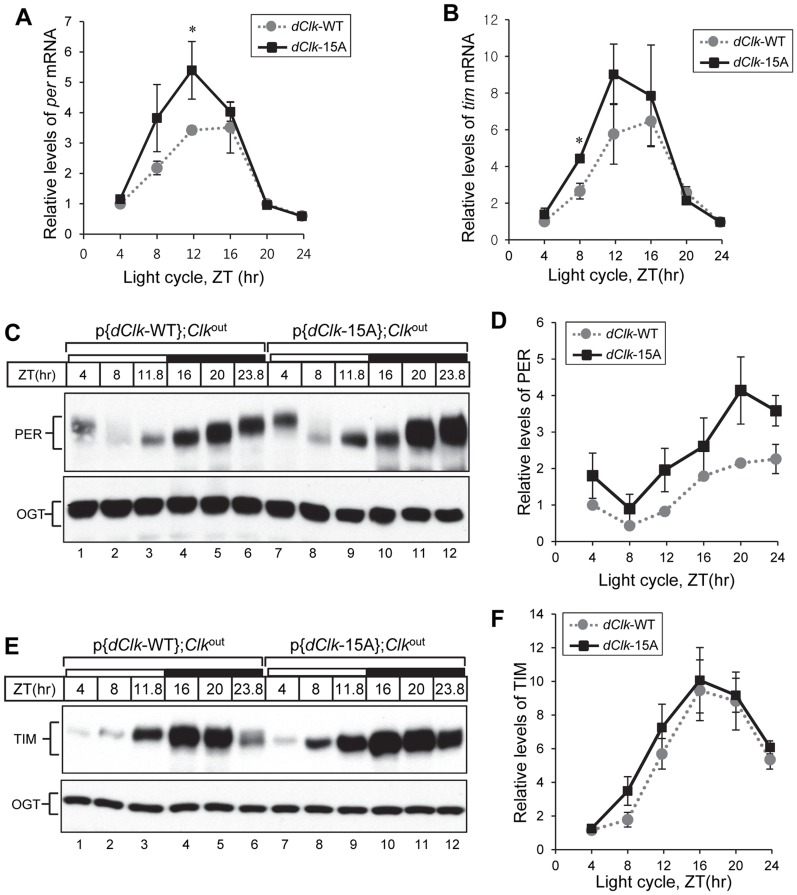
Higher amplitude rhythms of *per* mRNA and protein in p{*dClk*-15A};*Clk* ^out^ flies. Adult flies of the indicated genotype were collected at the indicated times (ZT) during a day and total RNA (A, B) or protein extracts (C to F) prepared. (A, B) Quantitative real-time RT-PCR was performed to measure the relative levels of *per* (A) or *tim* (B) transcripts. Shown are the average values from three independent experiments. (C to F) Immunoblotting was performed using anti-PER (Rb1) or anti-TIM (TR3) Ab. *O*-GlcNAc transferase (OGT) served as a loading control. Relative levels of PER and TIM proteins were determined by measuring band intensities of immunoblot using image J software (D, F). Shown are the average values from three independent experiments using p{*dClk*-15A}, 6M;*Clk* ^out^ flies. *p<0.05; error bars denote SEM.

To further examine the status of the clockworks, we measured the daily profiles in *per* and *tim* transcripts and protein levels. Both *per* and *tim* mRNA levels in p{*dClk*-15A};*Clk* ^out^ flies were reproducibly higher, especially during the daily upswing that occurs between ZT4 – 12 (Figure 4A and B). These result further support the notion that dCLK levels are normally rate-limiting for circadian transcription and suggest that despite the increased abundance of dCLK-15A there is sufficient PER to engage in normal repression of dCLK-15A/CYC activity. Indeed, PER protein levels were reproducibly higher in p{*dClk*-15A};*Clk* ^out^ flies (Figure 4C and D), consistent with the increased transcript levels. In p{*dClk*-15A};*Clk* ^out^ flies, TIM protein levels were slightly but reproducibly increased (Figure 4E and F). The increased daily upswing in *tim* mRNA levels in p{*dClk*-15A};*Clk* ^out^ flies might have a smaller effect on overall TIM protein levels because light induces the rapid degradation of TIM [47], possibly limiting the ability of TIM to accumulate during the early night prior to the start of transcriptional feedback repression. Taken together, we show that the stability of dCLK in flies is strongly increased by blocking phosphorylation at one or more sites. Moreover, augmenting the total abundance of dCLK accelerates the daily accumulation of *per*/*tim* transcripts and increases their peak levels, indicative of higher overall dCLK-CYC-mediated transcription. In addition, increased *in vivo* transcriptional activity of dCLK-15A may also contribute to higher dCLK-CYC-mediated transcription, as is the case in S2 cells (Figure 1D). These results demonstrate that dCLK phosphorylation plays a key role in setting the amplitudes of the *per* mRNA and protein rhythms, molecular oscillations that are central to the primary TTFL and circadian speed control in *Drosophila* [16], [48].

### Flies expressing dCLK-15A manifest a defect in behavioral synchronization to daily temperature cycles

**Figure 5:**
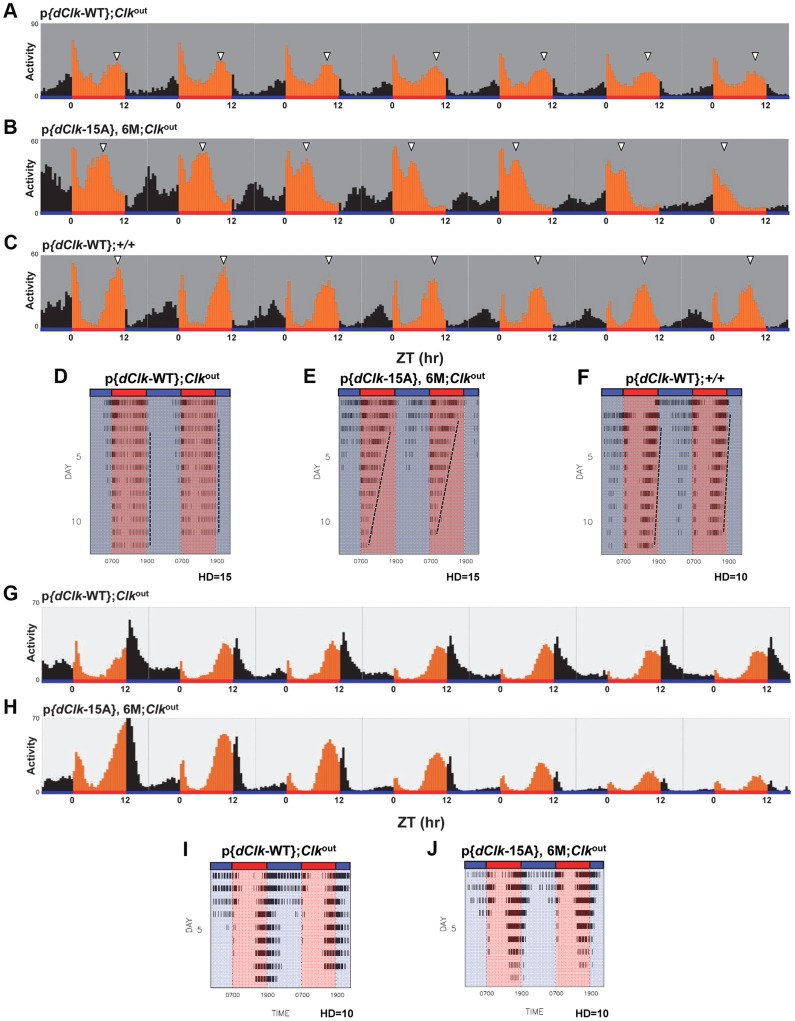
Impaired behavioral entrainment of p{*dClk*-15A};*Clk* ^out^ flies in daily temperature cycles. Adult male files of the indicated genotype were entrained in 12 h∶12 h temperature cycles of 24°C∶29°C in the absence (A–F) or presence (G–J) of constant light. (A–C, G, H) Each panel represents the daily average activity beginning on the third day of TC followed by 7 consecutive days. Orange vertical bars represent locomotor activities during the thermo phase and black vertical bars represent locomotor activities during the cryo phase. Red and blue horizontal bars indicate thermo- and cryo-phases, respectively. (D–F, I, J) Red and blue shades indicate thermo-and cryo-phases, respectively. The vertical black bars on each row of the actogram depict fly activity (measured in 30 min intervals). HD, hash density of the actogram (for example, HD = 10 signifies that 10 activity events are required to produce a hash mark). To better visualize rhythmic behavior, each row of an actogram was double plotted. To better visualize the progressive advancement of the main activity bout in p{*dClk*-15A};*Clk* ^out^ flies, a vertical line was drawn across the activity offsets.

Besides light-dark cycles, daily changes in temperature can also synchronize (entrain) circadian rhythms in a wide variety or organisms [49]. Prior work showed that *D. melanogaster* can entrain to daily cycles of alternating 12 hr ‘warm’/12 hr ‘cold’ cycles that differ by as little as 2–3°C [50]–[52]. To determine if flies expressing dCLK-15A have a defect in entraining to temperature cycle, flies were kept in constant darkness, exposed to 12 hr∶12 hr temperature cycles of 24°C∶29°C (TC) and locomotor activity rhythms analyzed (Table 3 and Figure 5). The daily distribution of activity in p{*dClk*-15A};*Clk* ^out^ flies is strikingly different compared to the wild-type control. As previously observed for wildtype strains of *D. melanogaster* entrained to daily temperature cycles [50]–[52], p{*dClk*-WT};*Clk* ^out^ flies exhibit the classic “anticipatory” rise in activity just prior to the low-to-high and high-to-low temperature transitions, similar to what is observed in light-dark cycles around ZT0 and ZT12 (Figure 5 and S5; there is a “startle” response at the transition from low-to-high temperature that is also observed in *Clk* ^out^ flies, analogous to the transient burst in activity at lights-on in a LD cycle). In sharp contrast, during the beginning of the temperature entrainment regime although p{*dClk*-15A};*Clk* ^out^ flies also manifest two activity peaks, they occur much earlier at around the middle of the warm- and cryo-phases (Figure 5B).

**Table 3:** Behavioral analysis of p{*dClk*-15A}; *Clk* ^out^ flies following temperature cycles.^a^

Genotype	Number^b^	Tau ± S.E.M. (h)	Rhythmicity (%)^c^	Power^d^
p{*dClk*-WT}, A;*Clk* ^out^	38	23.7±0.12	73.7	122.9
p{*dClk*-15A}, 2M; *Clk* ^out^	8	21.9±0.13	50	83.4
p{*dClk*-15A}, 6M; *Clk* ^out^	14	22.0±0.27	35.7	69.5
p{*dClk*-WT}, A;+/+	37	23.1±0.67	59.5	72.2

a Flies were kept in constant darkness and exposed to 12 hr∶12 hr temperature cycles of 24°C∶29°C for 9 days and followed by 7 days of constant 24°C.

b Total number of flies that survived until the end of the testing period.

c Percentage of flies with activity rhythms having a power value of ≥10 and a width value of ≥2.

d Measure of the strength or amplitude of the rhythm.

Interestingly, while the timing of the “startle” response at the transition from low-to-high temperature remained constant in p{*dClk*-15A};*Clk* ^out^ flies, the timing of the major activity peak occurring during the mid-warm phase appeared to progressively advance on subsequent days in TC (Figure 5B). Analysis of individual activity records also confirmed this trend (Figure 5E). The abnormal behavioral pattern under temperature cycles for p{*dClk*-15A};*Clk* ^out^ flies was also observed when flies were exposed to a temperature cycle after first treating them with constant light for 6 days to abolish the circadian timing system (Figure S6). Thus, the defective entrainment of p{*dClk*-15A};*Clk* ^out^ flies to TC is not dependent on the status of the clock at the time that the temperature entrainment was initiated. Furthermore, although the main activity bout in p{*dClk*-15A};*Clk* ^out^ flies advanced on subsequent days during TC, the rate of advancement was clearly greater during free-running conditions following TC (Figure S5), suggesting partial entrainment during TC. Following entrainment to TC, the free-running period of dCLK-15A producing flies is about 1.5 hr shorter compared to the wild type dCLK-WT control (Table 3 and Figure S5). The faster running clock in p{*dClk*-15A};*Clk* ^out^ flies during free-running conditions after exposure to TC is consistent with results obtained following entrainment to LD (Table 2). Thus, it appears that when exposed to daily temperature cycles p{*dClk*-15A};*Clk* ^out^ flies can adopt some alignment with the entraining conditions, albeit without a normal phase relationship, but that this entrainment is weak and the flies partially free-run at their shorter endogenous periods, leading to progressive advances in their behavioral rhythm relative to the 24 hr entraining regime. Although not as dramatic, the timing of the warm-phase activity bout in p{*dClk*-WT};+/+ flies also advanced during TC (Figure 5F), whereas this was not the case for p{*dClk*-WT};*Clk* ^out^ flies (Figure 5D). In addition, the clock in p{*dClk*-WT};+/+ runs about 1 hr faster than the control situation, strongly suggesting that augmenting dCLK levels (Figure S7) impairs the ability of the circadian timing system to entrain to daily temperature cycles.

Temperature cycles can entrain behavioral rhythms in *Drosophila* exposed to constant light (LL) despite the fact that LL normally abolishes circadian rhythms [51], [53], [54]. Intriguingly, constant light exposure rescues the ability of the p{*dClk*-15A};*Clk* ^out^ flies to maintain a more stable 24-hr phase relationship with the temperature cycle (Figure 5, panels G–J), further supporting the notion that entrainment to temperature but not light is specifically impaired in these flies. Taken together, these data suggest that in the absence of light, the dCLK phosphorylation program is required for the proper entrainment of behavioral rhythms to daily temperature cycles and reveal an unanticipated role for a central clock transcription factor in modality-specific entrainment.

### Molecular oscillations in p{*dClk-15A*};*Clk* ^out^ flies differ from those in control flies after prolonged exposure to temperature cycles

**Figure 6:**
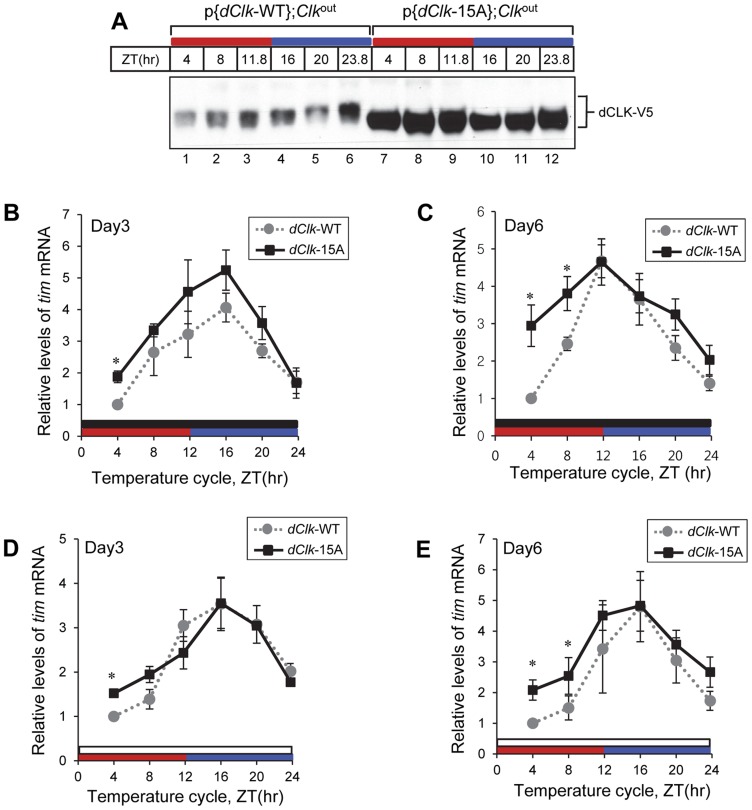
Molecular rhythms in p{*dClk*-15A};*Clk* ^out^ flies show increased alterations after prolonged entrainment to temperature cycles. Adult flies of the indicated genotype were entrained in 12 hr∶12 hr of 24°C∶29°C temperature cycle in the absence (A–C) or presence of light (D, E). During the third (A, B, D) and sixth day (C, E) of TC, flies were collected and protein (A) or RNA (B–E) was extracted from fly heads. Protein extracts were analyzed by immunoblotting using anti-V5 Ab to probe dCLK. Quantitative real-time RT-PCR was performed to measure the relative levels of *tim* mRNA. Shown are the average values from three independent experiments using p{*dClk*-15A}, 6M;*Clk* ^out^ flies. *p<0.05; error bars denote SEM. Red horizontal bars represent thermo phase, blue horizontal bars represent cryo phase, black horizontal bars represents constant dark conditions, and white horizontal bars represents constant light conditions.

In p{*dClk*-WT};*Clk* ^out^ flies, hypo/intermediate-phosphorylated dCLK isoforms are present throughout the thermo phase in TC (Figure 6A, lane 2 and 3), while hyper-phosphorylated dCLK isoforms are only observed during the latter half of the cryo phase (Figure 6A, lane 5 and 6). This temporal pattern in dCLK phosphorylation is similar to that observed in LD cycles and is consistent with prior work showing that the circadian clock mechanism in *Drosophila* can be synchronized by daily temperature cycles [50], [51]. As expected and similar to results using LD cycles, dCLK-15A attains higher overall daily levels and does not exhibit significant phosphorylation in p{*dClk*-15A};*Clk* ^out^ flies exposed to temperature cycles (Figure 6A).

Since p{*dClk*-15A};*Clk* ^out^ flies display altered entrainment to TC cycles that becomes progressively more abnormal with prolonged duration, we tested whether the molecular clock might also exhibit a more defective status with increasing time by measuring the levels of the *tim* mRNA at both early (e.g., day 3) and later (e.g., day 6) days of exposure to TC. We chose *tim* mRNA levels as a surrogate marker for clock dynamics because it normally has a robust high amplitude rhythm (Figure 4A, B; [55]), facilitating measuring changes in molecular oscillations over the course of several days. Although the daily average levels in *tim* mRNA were higher in p{*dClk*-15A};*Clk* ^out^ flies on day three of TC compared to the wild-type situation (Figure 6B), consistent with findings in LD (Figure 4B), both genotypes showed similar and robust cycling patterns. However, by day six of TC, the *tim* mRNA oscillation pattern in p{*dClk*-15A};*Clk* ^out^ flies became significantly different from that observed for p{*dClk*-WT};*Clk* ^out^ flies (Figure 6C). Most notably, while *tim* mRNA cycling still manifested high-amplitude cycling in p{*dClk*-WT};*Clk* ^out^ flies on day six of TC, *tim* mRNA levels during the upswing phase were significantly higher in p{*dClk*-15A};*Clk* ^out^ flies, resulting in an abnormal cycling pattern. Although we did not establish a causal relationship between the observed loss in normal *tim* mRNA cycling and the defective behavioral entrainment in p{*dClk*-15A};*Clk* ^out^ flies during TC, the results clearly show that prolonged exposure to TC is not only associated with increasingly altered phasing of rhythms at the behavioral level (Figure 5) but also at the molecular level.

As with behavioral rhythms prior work showed that circadian molecular cycles can be synchronized to TC in the presence of constant light [51], [53]. In agreement with the observation that constant light exposure enabled p{*dClk*-15A};*Clk* ^out^ flies to more robustly synchronize to temperature cycles (Figure 5), daily rhythms in the levels of *tim* mRNA for both genotypes were quite similar even after six days in constant light during TC (Figure 6D and E), indicating the clock in p{*dClk*-15A};*Clk* ^out^ flies is functioning in a more wild-type manner under these conditions. Taken together, while this molecular analysis is of limited scope, it suggests that constant light exposure facilitates the ability of p{*dClk*-15A};*Clk* ^out^ flies to entrain to TC by enhancing normal clock function.

## Discussion

Phosphorylation of clock proteins plays diverse roles in circadian oscillatory mechanisms by regulating numerous aspects of clock protein metabolism/activity, including time-of-day dependent changes in stability, transcriptional activity and subcellular localization [10]–[12]. Although dCLK, the master transcription factor in the *Drosophila* circadian clock [43], [56], undergoes daily changes in phosphorylation, the physiological role of dCLK phosphorylation was not clear. As a means to address this issue, we first identified phosphorylation sites on dCLK purified from cultured *Drosophila* S2 cells (Table 1 and Figure 1A). To examine the *in vivo* significance of dCLK phosphorylation, we generated transgenic flies expressing dCLK-15A wherein 15 serine residues that were identified as sites (or possible sites) of phosphorylation were switched to alanine, and examined circadian behavior in a *Clk* ^out^ genetic background. Our results indicate that global phosphorylation of dCLK is an important aspect of setting clock speed by regulating the daily levels and/or activity of dCLK. This is consistent with earlier work suggesting dCLK is the rate-limiting component in the central transcriptional/translational feedback loop (TTFL) in the *Drosophila* clock mechanism, and that increasing the levels of dCLK lead to shorter behavioral periods [5], [42], [43]. A surprising finding is that entrainment to daily temperature cycles but not light-dark cycles are highly dependent on dCLK phosphorylation. These results suggest a novel role for phosphorylation in circadian timing systems; namely, the effective strength of an entraining cue can be modulated by adjusting the dynamics of the TTFL via controlling the levels/activity of a master circadian transcription factor (see below).

In this study, we show that dCLK undergoes multi-site phosphorylation. Among the phospho-sites identified, seven serine residues are situated immediately N-terminal to a proline, indicating a major role for the CMGC group of kinases. Indeed, studies using cultured S2 cells suggested that dCLK is a potential target of several distinct CMGC kinases [32]. More recent work also identified the pro-directed kinase NEMO as a dCLK-relevant kinase [27]. Ongoing work is aimed at identifying the kinases responsible for targeting the different phospho-sites on dCLK. It should be noted that in this study we mapped phosphorylation sites on dCLK expressed in S2 cells, which when resolved by SDS-polyacrylamide gel electrophoresis is mainly observed as two major electrophoretic mobility bands corresponding to non/hypo-phosphorylated isoforms and an ‘intermediate’ more highly phosphorylated slower migrating species [29]. Although DBT is endogenously expressed in S2 cells, the addition of exogenous DBT and/or the inhibition of protein phosphatases leads to the detection of hyper-phosphorylated isoforms of dCLK in S2 cells [29]. Thus, it is likely that we did not identify all the phospho-sites on dCLK. However, we cannot rule out the possibility that there were minor levels of hyper-phosphorylated dCLK in our preparations that were above the detection limit for phospho-site mapping by mass spectrometry. Irrespective, the phospho-sites that we identified in S2 cultured cells make a clear contribution to the daily dCLK phosphorylation program in flies and contribute to the circadian timing system.

Elimination of phosphorylation sites from dCLK (dCLK-15A) leads to significant increases in the overall daily levels of dCLK in flies, which is well correlated with previous reports in S2 cells showing that hyper-phosphorylated dCLK is sensitive to degradation [28], [29]. In general, global phosphorylation appears to reduce the stabilities of clock proteins by generating one or more phospho-degrons that are recognized by E3 ubiquitin ligases, which ultimately leads to the accelerated degradation of the phosphorylated isoforms via the proteasome pathway [13]. The E3 ligase termed CTRIP appears to directly regulate the levels of dCLK (and possibly PER), although the role of dCLK phosphorylation in this mechanism, if any, is not clear [57]. When assayed in S2 cells the stability of dCLK-16A was similar to that of dCLK-WT (e.g., Figure 1 and Figure S2). Because differences in transcript levels cannot explain the significantly higher levels of dCLK-15A in flies compared to dCLK-WT (Figure 3), it is almost certain that dCLK-15A is a more stable protein in clock cells. Thus, it appears that S2 cells do not fully recapitulate the *in vivo* role of phosphorylation on dCLK degradation. If we did miss mapping some sites on hyper-phosphorylated dCLK that are critical for regulating stability it is possible that these sites can still be phosphorylated on dCLK-15A expressed in S2 cells but not in flies. For example, hyper-phosphorylation of dCLK might depend on prior phosphorylation at one or more of the 15 phospho-sites we identified, and this dependency might be more strict in flies compared to the S2 cell over-expression system. Hierarchical phosphorylation has been demonstrated for other clock proteins, such as *Drosophila* PER and mammalian CLK [15], [37], [40]. Future work will be required to determine if there are other phospho-sites besides those we identified that regulate dCLK stability in flies.

Besides regulating the stability of core clock transcription factors, phosphorylation modulates trans-activation potential [36], [37], [58]–[61]. dCLK-15A expressed in S2 cells exhibited normal binding to CYC (and PER) but exhibits more potent transcriptional activity, at least in the context of a simple E-box driven expression (Figure 1D). Consistent with this, the levels of *dper* and *tim* mRNAs in p{*dClk*-15A};*Clk* ^out^ flies are higher compared to the control situation (Figure 4A, B). Of course, phosphorylation also affects the levels of dCLK-15A in flies, so at this stage it is not possible to determine how much the increased *per*/*tim* transcript levels are due to changes in the levels or activity of dCLK-15A. Nonetheless, our results strongly suggest that in wild-type flies the levels and/or activity of dCLK act in a rate-limiting fashion during the daily accumulation phase of *per/tim* transcripts and possibly other targets. In addition, the phospho-sites that we identified do not seem to be play a major determinant in feedback repression by PER and associated factors. Strong repression was observed in S2 cells for the dCLK-15A version (Figure 1E) and the normal daily downswing in *per*/*tim* levels occurred in p{*dClk*-15A};*Clk* ^out^ flies (Figure 4A and B). However, it is possible that we missed some phospho-sites that more specifically regulate the transcriptional activity of dCLK.

At the behavioral level, p{*dClk*-15A};*Clk* ^out^ flies exhibit short period rhythms, consistent with prior work showing that increasing the dosage of *dClk* quickens the pace of the clock [42], [43]. In light-dark cycles, p{*dClk*-15A};*Clk* ^out^ flies maintain a stable phase relationship with the entraining environment, displaying the typical anticipatory bimodal activity pattern (Figure 2). Moreover, in a daily light-dark cycle the timing of the morning and especially evening peak of activity is shifted in flies with different endogenous periods, appearing earlier in fast clocks and later in slow clocks [52]. Indeed, the p{*dClk*-15A};*Clk* ^out^ flies follows this trend as the evening (and morning) bout of activity in LD is earlier compared to control flies (Figure 2). Together, these results indicate that although global phosphorylation of dCLK is an important determinant in setting clock speed, it plays little to no role in photic entrainment.

Surprisingly, the elimination of phosphorylation sites on dCLK strongly influences circadian behavior in daily temperature cycles (Figure 5). Temperature cycles with amplitudes of only 2° to 3°C robustly synchronizes circadian rhythms in *Drosophila* and other organisms [51], [52], [62]–[66]. When exposed to temperature cycles of 24°C/29°C, control p{*dClk*-WT};*Clk* ^out^ flies manifested the typical bimodal activity pattern with bouts of activity anticipating the two temperature transition points, similar to that occurring during entrainment to LD cycles (Figure 5A and S5A). However, even during the first days in TC, p{*dClk*-15A};*Clk* ^out^ flies already exhibit a very abnormal phase alignment with ‘morning’ and ‘evening’ bouts of activity that occur much earlier, around the middle of the cryo- and thermal-phases, respectively (Figure 5B and S5B, C). The advanced timing of the morning and evening bouts of activity is much earlier than would be expected based solely on the 1.5 hr shorter circadian period in p{*dClk*-15A};*Clk* ^out^ flies (Table 3). That entrainment to TC is highly defective in p{*dClk*-15A};*Clk* ^out^ flies is even more dramatically underscored by the progressive advances in the evening component of activity on subsequent days (Figure 5). Although not as apparent, flies with increased dosage of *dClk* (p{*dClk*-WT};+/+ flies) also showed progressively earlier evening activity bouts in thermal cycles (Figure 5C and F) but not LD cycles, further suggesting that increased levels/activity of dCLK are causally linked to the inability of maintaining a stable phase relationship with TC. Because the timing of the evening activity in both p{*dClk*-15A};*Clk* ^out^ and p{*dClk*-WT};+/+ flies occurs progressively earlier during TC, our results strongly suggest that these flies are only weakly synchronized to TC and are partially free-running at their faster endogenous periods.

In trying to determine why p{*dClk*-15A};*Clk* ^out^ flies might exhibit a defect in temperature entrainment but not photic entrainment, it is important to note that several lines of evidence support the notion that light is a more potent synchronizer of the clock in *D. melanogaster* compared to temperature entrainment, including the use of out-of-phase light/dark and temperature cycles [4]. In addition, lowering the levels/function of the key photic entrainment photoreceptor CRYPTOCHROME (CRY) increases the ability to synchronize to TC [67], suggesting the dominance of light input under normal conditions. Also, it takes many more days to shift the phase of the clock via TC compared to LD cycles [50]. The overall strength of light in *D. melanogaster* entrainment is not surprising given the ability of light pulses to evoke the rapid degradation of TIM and the great sensitivity of *Drosophila* CRY/TIM to light [68].

Indeed, constant light rescues the ability of TC to stably entrain behavioral rhythms in p{*dClk*-15A};*Clk* ^out^ (Figure 5, G–J), presumably by maintaining the clock in a more normal state (Figure 6). Intriguingly, prior work showed a similar pattern for the classic *per* ^S^ and *per* ^L^ mutants that display short (19 hr) and long (29 hr) endogenous rhythms, respectively [66]. That is, while wild-type flies entrain to TC in DD or LL, but *per* ^S^ and *per* ^L^ flies only entrain to TC in LL [66]. This suggests that alterations in the PER protein rhythm might preferentially disrupt thermal entrainment. In the case of p{*dClk*-15A};*Clk* ^out^ flies the amplitude of the PER abundance cycle is increased reaching higher peak values (Figure 4). Clocks with higher amplitudes are more resistant to entrainment by weak zeitgebers [69]–[71]. Relevant to this discussion, reducing CLOCK activity in mice decreased the amplitude of the circadian pacemaker and *per* gene expression, enhancing the ability to evoke phase shifts in behavioral rhythms [72], [73]. Thus, a simple model for our results is that the increased *per* mRNA and protein rhythms in p{*dClk*-15A};*Clk* ^out^ flies leads to an increase in pacemaker amplitude minimizing their ability to synchronize to weaker entraining signals such as TC. However, it should be noted that higher amplitude rhythms of cycling mRNAs are highly suggestive but not definitive proof of an increase in oscillator. A standard approach to infer the relative amplitude of a clock is to increase the strength of the entraining signal, which should enhance its entrainment potential [69], [70], [74].

Although a change in the amplitude of the clock in p{*dClk*-15A};*Clk* ^out^ flies offers a plausible explanation for the preferential defect in temperature entrainment, there are other possibilities. For example, CRY-positive clock cells are more important for entraining to LD cycles, whereas CRY-negative clock cells are more important for TC entrainment [4]. Thus, dCLK-15A could have preferential effects in CRY-negative cells to lessen their contribution, impairing TC entrainment. Another more speculative idea is that the phosphorylation of dCLK can act as a thermal sensor, although this would be specific to temperature entrainment as temperature compensation appears normal in the p{*dClk*-15A};*Clk* ^out^ flies (Table 2). Clearly, future studies will be required to better address the mechanism underlying the impaired synchronization of p{*dClk*-15A};*Clk* ^out^ flies to temperature cycles. However, our findings reveal that phosphorylation of a key rate-limiting circadian transcription factor is critical for entrainment to daily temperature cycles. Indeed, the CLOCK protein in zebrafish [65] was shown to be regulated by temperature, suggesting a universal role for CLOCK in the adaptation of animal circadian clocks to thermal cues.

## Materials and Methods

### Plasmids for tissue culture

The pMT-*dClk*-V5, pMT-HA-*dClk*-V5, pMT-HA-*dClk*, pAct-*per*, pAct-*per*-V5 and pMT-*dbt*-V5 plasmids were described previously [20], [29], [31]. pMT-*dClk*15A-V5 and pMT-*dClk*16A-V5 were generated by serially changing codons for Ser to those of Ala by using a Quick Change site-directed mutagenesis kit (Stratagene). All final constructs were verified by DNA sequencing.

### Identification of dCLK phosphorylation sites by mass spectrometry

Hygromycin-resistant stable Schneider 2 (S2) cell lines expressing pMT-HA-*dClk*-V5 were established for dCLK purification. *dClk* expression was induced by adding 500 µM CuSO_4_ to the medium and cells were harvested 24 hr post-induction. 200 ml of culture (3×10^6^ cells/ml) was used and harvested cells were lysed using modified-RIPA buffer (50 mM Tris-HCl [pH 7.5], 150 mM NaCl, 1% NP-40, 0.25% Sodium deoxycholate) with the addition of a protease inhibitor cocktail (Roche) containing 1 mM EDTA, 25 mM NaF, and 1 mM Na_2_VO_3_. To extracts, anti-V5 antibody (Invitrogen) was added and incubated overnight with gentle rotation at 4°C followed by the addition of Dynabeads Protein A (Invitrogen) with a further overnight incubation. Beads were collected using DynalMPC. dCLK was eluted with modified Laemmli buffer (150 mM Tris-HCl [pH 6.8], 6 mM EDTA, 3% SDS, 30% Glycerol) supplemented with 50 mM reducing agent TCEP (Calbiochem) at 65°C for 20 min. Alkylation was performed by adding 0.5M IAA (iodoacetamide) for 20 min at room temperature in the dark. The eluate was resolved using 8% SDS-PAGE, and all the detectable dCLK bands of differing electrophoretic mobility excised (which under the conditions used was mainly the ‘intermediate’ phosphorylated band), subjected to protease digestion and analyzed by mass spectrometry. Mass spectrometry was performed as described in Schlosser et al. 2005. Data analysis was performed as described previously [41].

### Luciferase assay

S2 cells were obtained from Invitrogen and transfected using effectene following the manufacturer's protocol (Qiagen). *Luciferase* (*luc*) reporter assay was performed as described previously [29], [75]. Briefly, S2 cells were placed in 24-well plates and co-transfected with 0–100 ng pMT-*dClk*-V5 and pMT-*dClk*-16A-V5 along with 10 ng of perEluc, 30 ng of pAct-*β-gal*-V5/His as indicated. dPER mediated repression of dCLK dependent transactivation was measured by transfecting 0–20 ng of pAct-*dper* together with 2 ng of pMT-*dClk*-V5 or pMT-*dClk*-16A-V5. One day after transfection, *dClk* expression was induced with 500 µM CuSO_4_ (final in the media), and after another day cells were washed in phosphate buffered saline (PBS), followed by lysis in 300 µl of Reporter Lysis Buffer (Promega). Aliquots of cell extracts were assayed for β-galactosidase and luciferase activities using the Luciferase Assay System and protocols supplied by the manufacturer (Promega).

### Transgenic flies

*Clk* ^out^ flies were generated in one of our laboratories (P.E.H.) as follows: 5.2 kb deletion of *dClk* exon 1 and upstream sequences was generated by FLP-mediated recombination between FRT sites in the pBac Clk[f06808] and pBac Clk [f03095] [76], [77]. Flippase (FLP)-induced recombination was induced by a daily 1 h heat-shock at 37°C given to hsFLP;;f06808/f03095 larvae and pupae. Three recombinants were recovered, and each produced a deletion rather than a duplication of intervening *dClk* sequences. The remaining pBac insert was excised via pBac transposase induced transposition resulting in white-eyed flies harboring the deletion [78]. A DNA fragment containing the deleted sequences was amplified using primers situated upstream of the f03095 insertion site (5′ CGGAATATTGGACAACAAACAG 3′) and downstream of the f06808 insertion site (5′CAGCAGTGGAATCTTAATACAG 3′), and sequenced to confirm the endpoints of the deletion. This new *dClk* deletion allele was named *Clk* ^out^.

To generate transgenic flies that produce wild-type dCLK tagged with V5 at the C-terminus, *dClk*-containing P[acman] transgene was generated using recombineering-mediated gap repair [79]. To prepare the P[acman] vector, homology arms were amplified from genomic DNA with primers clkLA-f (ATGTGGCGCGCCGCCCCAAAAATCCATAAATGCT) and clkLA-r (GTGTTGGATCCAGGGGTGTTATAGAGAGGGACA) for the left arm and clkRA-f (GTGTGGATCCGCAGAGTGAAACCTGTGCAA) and clkRA-r (ATATATGTGCGGCCGCTCCCGGTTATGAGTTTTTCG) for the right arm via PCR, and cloned as AscI-BamHI and BamHI-NotI fragments into AscI and NotI digested attB-P[acman]-ApR vector (modified to remove the SphI site) to form attB-P[acman]ClkLARA. Recombination-competent SW102 cells harboring BAC clone RP98 5K6 (BACPAC Resource Center, Oakland, Ca, USA), which contains the *dClk* genomic region, were transformed with the attB-P[acman]ClkLARA vector (linearized with BamHI). Recombinants containing 15.5 kb of genomic sequence beginning ∼8 kb upstream of the *dClk* translation start and ending ∼2.5 kb downstream of the *dClk* stop codon were verified by PCR and sequencing and termed attB-P[acman]-*Clk*. To introduce a V5 epitope tag at the C-terminus of the *dClk* open reading frame (ORF), a 3′ genomic fragment of *dClk* (from 351 bp upstream to 1580 bp downstream of the translation stop) was cloned into pGEM-T vector (Promega, Madison, WI). Sequences encoding V5 were introduced in-frame immediately upstream of the *dClk* stop codon using the Quickchange site directed mutagenesis kit (Stratagene, La Jolla, CA) to create pGEM-T-*dClk*3′V5. The 3′ *dClk* genomic fragment in attB-P[acman]- *dClk* was swapped with the 3′ fragment in pGEM-T-*dClk*3′V5 using SphI and NotI to form attB-P[acman]- *dClk*V5. This transgene was inserted into the VK00018 attP site on chromosome 2 via PhiC31-mediated transgenesis [79], [80].

Transformation vector containing a genomic *dClk* wherein the codons for the 15 identified phospho-serine were switched to those for alanine was generated in multiple stages as follows: A genomic *dClk* sub-fragment from NheI to SphI site was isolated from P[acman]-*dClk*-V5 and subcloned into pSP72 vector where the multi-cloning sites were mutagenized to introduce a NheI site, and named this plasmid as pSP72-*dClk*(NheI/SphI). Next, we obtained a *dClk* sub-fragment spanning from the NcoI to SphI sites by restriction digestion of pSP72-*dClk*(NheI/SphI), subcloned the released fragment into pSP72 where the multi-cloning sites were mutagenized to introduce a NcoI site, and named this plasmid as pSP72-*dClk*(NcoI/SphI). We performed serial site directed mutagenesis with pSP72-*dClk*(NcoI/SphI) and finally made pSP72-*dClk*(NCoI/SphI)-S11A wherein codons for the serine residues at amino acids 209, 210, 211, 444, 450, 487, 504, 611, 645, 859, 902 on dCLK were all switched to those for alanine residues [(GenBank accession number NP_001014576)]. We purified the *dClk*(NCoI/SphI)-S11A insert by restriction enzyme digestion of pSP72-*dClk*(NCoI/SphI)-S11A and replaced the wild-type *dClk*(NcoI/SphI) insert, generating pSP72-*dClk*(NheI/SphI)-S11A. Next, a more 3′ genomic *dClk* sub-fragment from the SphI to NotI sites was subcloned into pSP72 vector where the multi-cloning sites were mutagenized to include NotI and NheI sites, and named this plasmid as pSP72-*dClk*(SphI/NotI). We performed serial site directed mutagenesis with pSP72-*dClk*(SphI/NotI) and made pSP72-*dClk*(SphI/NotI)-S4A wherein codons for the serine residues at amino acids 924, 934, 938, 1018 were switched to those for alanine. Finally, the genomic *dClk*(SphI/NotI)-S4A fragment was ligated with pSP72-*dClk*(NheI/SphI)-S11A generating pSP72-*dClk*(NheI/NotI)-S15A, and then *dClk*(NheI/NotI)-S15A fragment was switched with wild-type *dClk*(NheI/NotI) fragment in pacman-*dClk*-V5 plasmid yielding P[acman]-*dClk*-15A-V5. Transgenic flies were generated by BestGene Inc. (CA, USA). P[acman]-*dClk*-15A-V5 transformation vector was injected into flies carrying the VK00018 attP docking site on the second chromosome for site-specific integration [79]. Two independent germ-line transformants bearing the *dClk*-15A-V5 transgene in a wild-type background were obtained and then crossed into a *Clk* ^out^ genetic background to yield *dClk*-15A-V5;*Clk* ^out^.

### Behavioral assays

The locomotor activities of individual flies were measured as previously described using the Drosophila Activity Monitoring system from Trikinetics (Waltham, MA). Young adult flies were used for the analysis and exposed to 4 days of 12 h light followed by 12 h dark [where zeitgeber time 0 (ZT0) is defined as the time when the light phase begins] at 25°C and subsequently kept in constant dark conditions (DD) for 7 days. Temperature entrainment (temperature cycle, TC) was performed in constant dark condition and in some cases, in the presence of constant light (>2000lux). Temperature cycles were 12 h of 24°C (cryo phase) followed by 12 h of 29°C (thermal phase) (where ZT0 is defined as the time when the cryo phase begins) for 4 days and subsequently kept at 24°C for 7 days. The locomotor activity data for each individual fly was analyzed using the FaasX software (Fly Activity Analysis Suite for MacOSX), which was generously provided by F. Rouyer (CNRS, France). Periods were calculated for each individual fly using *chi*-square periodogram analysis and pooled to obtain a group average for each independent transgenic line or genotype. Power is a quantification of the relative strength of the rhythm during DD. Individual flies with a power ≥10 and a ‘width’ value of 2 or more (denotes number of peaks in 30-min increments above the periodogram 95% confidence line) were considered rhythmic. Actogram represents the locomotor activity data throughout the experimental period. Vertical bars in the actogram represent absolute activity levels for each 30 min intervals averaged for each given genotypes of flies. The strength of this measurement can be manipulated by using the function called hash density, which represent the number of times fly need to make beam crossing to be registered as one vertical bar. The hash density of the actogram was varied for better comparison depending on the activity levels of given genotypes of flies.

### Immunoblotting and immunoprecipitation

Protein extracts from S2 cells were prepared as previously described [31]. Briefly, the cells were lysed using modified-RIPA buffer (50 mM Tris-HCl [pH 7.5], 150 mM NaCl, 1% NP-40, 0.25% Sodium deoxycholate) with the addition of protease inhibitor cocktail (GeneDEPOT) and phosphatase inhibitor cocktail (GeneDEPOT). For detection of dCLK recombinant protein, extracts were obtained using RIPA buffer 25 mM Tris-HCl [pH 7.5], 50 mM NaCl, 0.5% Sodium deoxycholate, 0.5% NP40, 0.1% SDS) and were sonicated briefly as previously described [29]. Flies were collected by freezing at the indicated times in light-dark (LD) or temperature cycles (TC) and total fly head extracts prepared using modified-RIPA buffer or RIPA buffer with sonication (for dCLK). Extracts were resolved by 5% polyacrylamide gels or by 3–8% Tris-acetate Criterion gel (Bio-Rad) in some case for dCLK, transferred to PVDF membrane (Immobilon-P, Millipore), and immunoblots were treated with chemiluminescence (ECL, Thermo). Primary antibodies were used at the following dilutions; anti-V5 (Invitrogen), 1∶5000; anti-HA (12CA5, Roche), 1∶2000; anti-OGT (H-300, Santa Cruz), 1∶3000; anti-PER, (Rb1) 1∶3000; anti-TIM (TR3), 1∶3000; anti-dCLK (GP208) 1∶3000. Quantification of band intensity was performed using image J software.

For immunoprecipitation, cell extracts from S2 cells were prepared and 3 µl of anti-HA (12CA5) or anti-V5 antibody was added depending on the target protein sought, and incubated for overnight at 4°C with gentle rotation. The next day, 20 µl of Gammabind-sephase bead (GE healthcare) was added with a further incubation of 3 hr at 4°C. The immune complexes were eluted with 1X SDS-PAGE sample buffer. For λ- phosphatase treatment, the purified immune complexes were resuspended in λ protein phosphatase buffer (50 mM Tris-HCl [pH 7.5], 0.1 mM EDTA, 5 mM DTT, 0.01% Triton X-100, 2 mM MnCl_2_, and 0.1 mg/ml bovine serum albumin), divided into two equal aliquots. One aliquot of bead was treated with 200 units of λ protein phosphatase (NEB) and no addition was made to the other aliquot. Both aliquots were incubated for 30 min at 30°C with occasional shaking, and immune complexes analyzed by immunoblotting.

### Quantitative real time RT-PCR

Total RNA was isolated from frozen heads using QIAzol lysis reagent (QIAGEN). 1 µg of total RNA was reverse transcribed with oligo-dT primer using Prime Script reverse transcriptase (TAKARA) and real-time PCR was performed in Corbett Rotor Gene 6000 (Corbett Life Science) using Quantitect SYBR Green PCR kit (Qiagen). Primer sequences used here are as follows; *dper* forward: 5′-GACCGAATCCCTGCTCAATA-3′; *dper* reverse: 5′-GTGTCATTGGCGGACTTCTT-3′; *tim* forward: 5′-CCCTTATACCCGAGGTGGAT-3′; *tim* reverse: 5′-TGATCGAGTTGCAGTGCTTC-3′; *dClk* forward: 5′-CAGCCGCAATTCAATCAGTA-3′; *dClk* reverse: 5′-GCAACTGTGAGTGGCTCTGA-3′. We also included primers for the noncycling mRNA coding for CBP20 as previously described, and sequences are as follows; *cbp*20 forward: 5′-GTCTGATTCGTGTGGACTGG-3′; *cbp*20 reverse: 5′-CAACAGTTTGCCATAACCCC-3′. Results were analyzed with software associated with Rotor Gene 6000, and relative mRNA levels were quantitated using the 2^−ΔΔCt^ method.

## Supporting Information

Figure S1 Analysis of CLK phosphorylation site mutants for electrophoretic mobility and transcriptional activity in S2 cells (A, B) S2 cells were transiently transfected with 500 ng of wild-type (WT) or serine to alanine mutated version of pMT-HA-*dClk*-V5. Mutated sites are indicated on the top. Expression of dCLK was induced 24 hr after transfection by adding 500 µM CuSO_4_ to the medium. Cells were harvested 24 hr after induction, and protein extracts were subjected to western blot analysis. dCLK was visualized with anti-V5 Ab. Please note that the decrease in the levels of dCLK S859A (A) was not reproducible as shown in (B). (C) Shown are the average values for relative E box dependent luciferase activity in the presence of 2 ng of wild-type (WT) or serine to alanine mutated version of pMT-HA-*dClk*-V5. dCLK-S875A was included as randomly chosen serine to alanine mutant.
(TIF)

Figure S2 Stability of dCLK-WT and dCLK-16A protein in S2 cells. (A, B) S2 cells were transiently transfected with 300 ng of pMT-HA-*dClk* (WT) or pMT-HA-*dClk*-15A (15A) singly (A) or in combination with 600 ng of pMT-*dbt*-V5 (B). Expression of dCLK and DBT was induced 24 hr after transfection by adding 500 µM CuSO_4_ to the medium. 24 hrs post induction, 10 µg/ml of cycloheximide (CHX) was treated to inhibit translation. Cells were harvested at the indicated time points and protein extracts were subjected to immunoblotting. dCLK was visualized with anti-HA (3F10) antibody. Shown are the representative blots for each analysis and relative levels of dCLK proteins were determined by measuring band intensities of immunoblot using image J software.
(TIF)

Figure S3 dCLK-15A manifests similar alterations as dCLK-16A compared to dCLK-WT in terms of stability and transcriptional activity in S2 cells. (A) S2 cells were transiently transfected with 500 ng of pMT-HA-*dClk* (WT) or pMT-HA-*dClk*-15A (15A). Expression of dCLK was induced 24 hr after transfection by adding 500 µM CuSO_4_ to the medium. Cells were harvested 24 hr after induction and protein extracts were first subjected to immunoprecipitation using anti-HA (12CA5) antibody and immune complexes were incubated in the absence (−) or presence (+) of λ phosphatase followed by immunoblotting. (B) Shown are the average values from three independent experiments for relative E box dependent luciferase activity in the absence (BL) or presence of pMT-*dClk*-V5 (WT) or pMT-*dClk*-15A (15A).
(TIF)

Figure S4 (A–D) Representative daily locomotor activity patterns of p{*dClk*-WT};*Clk* ^out^ and p{*dClk*-15A};*Clk* ^out^ flies in light/dark cycles. Adult flies of the indicated genotype (as indicated, top of panels) were entrained with 12 hr∶12 hr light∶dark cycles for 4 days followed by 8 days in DD. Black and white bar on top of each actogram indicates when lights were off and on, respectively. Red arrowhead indicates when DD starts. The vertical black bars on each row of the actogram depict the activity of the fly (measured in 30 min intervals). To better visualize rhythmic behavior, each day's worth of activity recordings was double plotted. HD, hash density of the actogram.
(TIF)

Figure S5 (A–D) Representative daily locomotor activities of p{*dClk*-WT};*Clk* ^out^ and p{*dClk*-15A};*Clk* ^out^ flies in temperature cycles in the absence of light. Adult male files for a given genotype (as indicated, top of panels) were entrained in 12 hr∶12 hr temperature cycles of 24°C∶29°C for 9 days and maintained at 24°C for 7 days in the absence light. The vertical black bars on each line of the actogram depict fly activity (measured in 30 min intervals). Each day's worth of activity recordings was double plotted to better visualize rhythmic behavior. Red horizontal bars and blue horizontal bars below each panel indicated thermo- or cryo-phases, respectively. The results clearly indicate that the offset in evening activity occurs progressively earlier in p{*dClk*-15A};*Clk* ^out^ flies even during TC. HD, hash density of the actogram.
(TIF)

Figure S6 (A–C) Representative daily locomotor activities of p{*dClk*-WT};*Clk* ^out^ and p{*dClk*-15A};*Clk* ^out^ flies in temperature cycles after exposure to constant light. Adult male files for a given genotype (as indicated, top of panels) were exposed to constant light for 6 days and then entrained in 12 hr∶12 hr temperature cycles of 24°C∶29°C for 7 days in the absence light. The red arrowhead indicates when the lights were turned off. The vertical black bars on each line of the actogram depict fly activity (measured in 30 min intervals). Each day's worth of activity recordings was double plotted to better visualize rhythmic behavior. Flies became arrhythmic shortly after exposure to constant light. The results clearly indicate that the offset in evening activity occurs progressively earlier in p{*dClk*-15A};*Clk* ^out^ flies during TC. HD, hash density of the actogram.
(TIF)

Figure S7 dCLK protein levels and phosphorylation in p{*dClk*-WT};*Clk* ^out^ and p{*dClk*-WT};+/+ flies. Adult flies of a given genotype (indicated at the top of panels) were collected at the indicated time in LD (ZT) and protein extracts analyzed by immunoblotting using the anti-dCLK antibody (gp208). Note that the levels of dCLK are higher in p{*dClk*-WT};+/+ flies.
(TIF)
