# Supplementary figures and images for: Phosphorylation of a Central Clock Transcription Factor Is Required for Thermal but Not Photic Entrainment
Source: PLoS Genet. 2014 Aug 14;10(8):e1004545. doi: 10.1371/journal.pgen.1004545 (PMC4133166; doi:10.1371/journal.pgen.1004545)

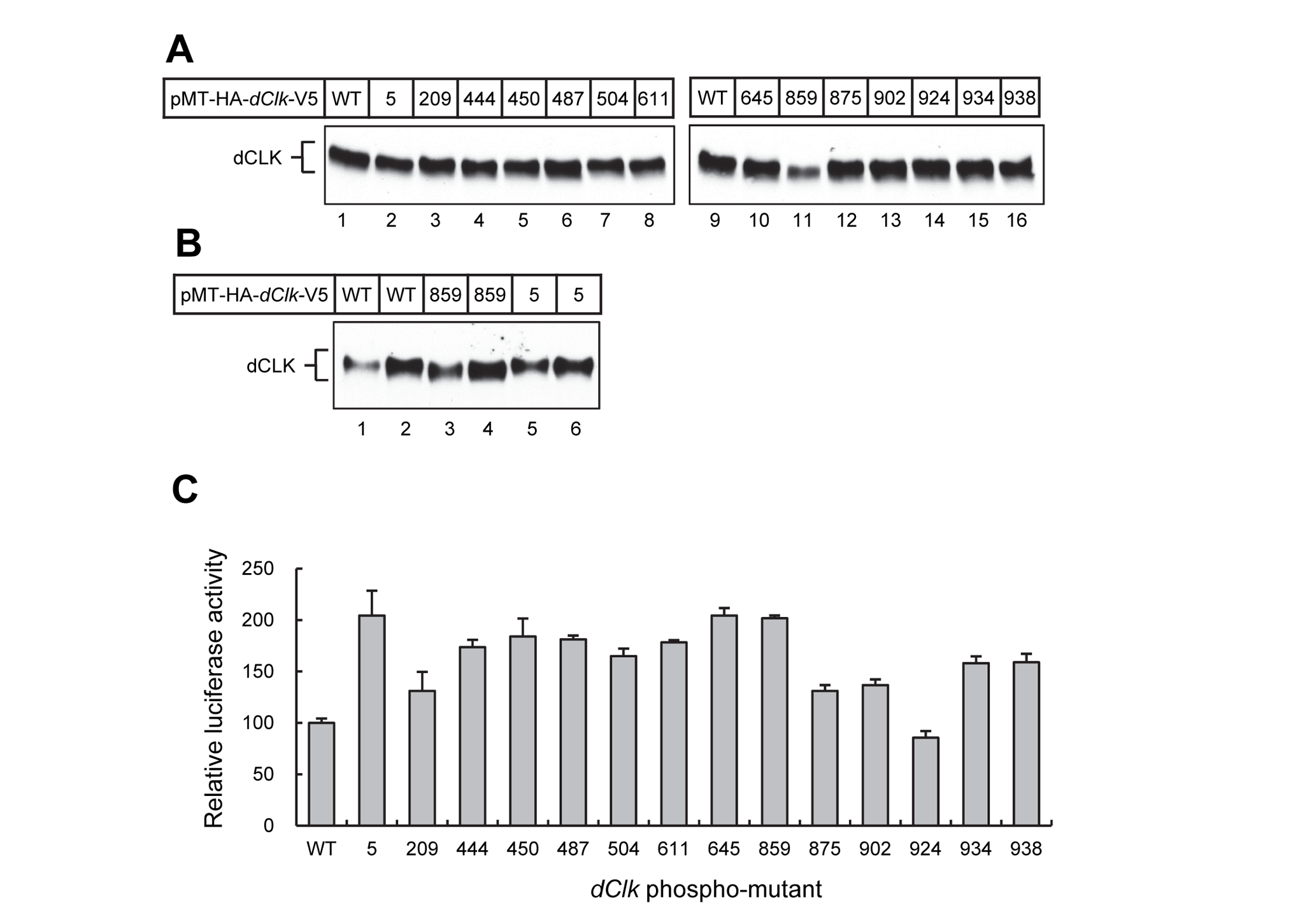

Supplement: Figure S1 — Analysis of CLK phosphorylation site mutants for electrophoretic mobility and transcriptional activity in S2 cells (A, B) S2 cells were transiently transfected with 500 ng of wild-type (WT) or serine to alanine mutated version of pMT-HA-dClk-V5. Mutated sites are indicated on the top. Expression of dCLK was induced 24 hr after transfection by adding 500 µM CuSO4 to the medium. Cells were harvested 24 hr after induction, and protein extracts were subjected to western blot analysis. dCLK was visualized with anti-V5 Ab. Please note that the decrease in the levels of dCLK S859A (A) was not reproducible as shown in (B). (C) Shown are the average values for relative E box dependent luciferase activity in the presence of 2 ng of wild-type (WT) or serine to alanine mutated version of pMT-HA-dClk-V5. dCLK-S875A was included as randomly chosen serine to alanine mutant. (TIF) [file pgen.1004545.s001.tif]

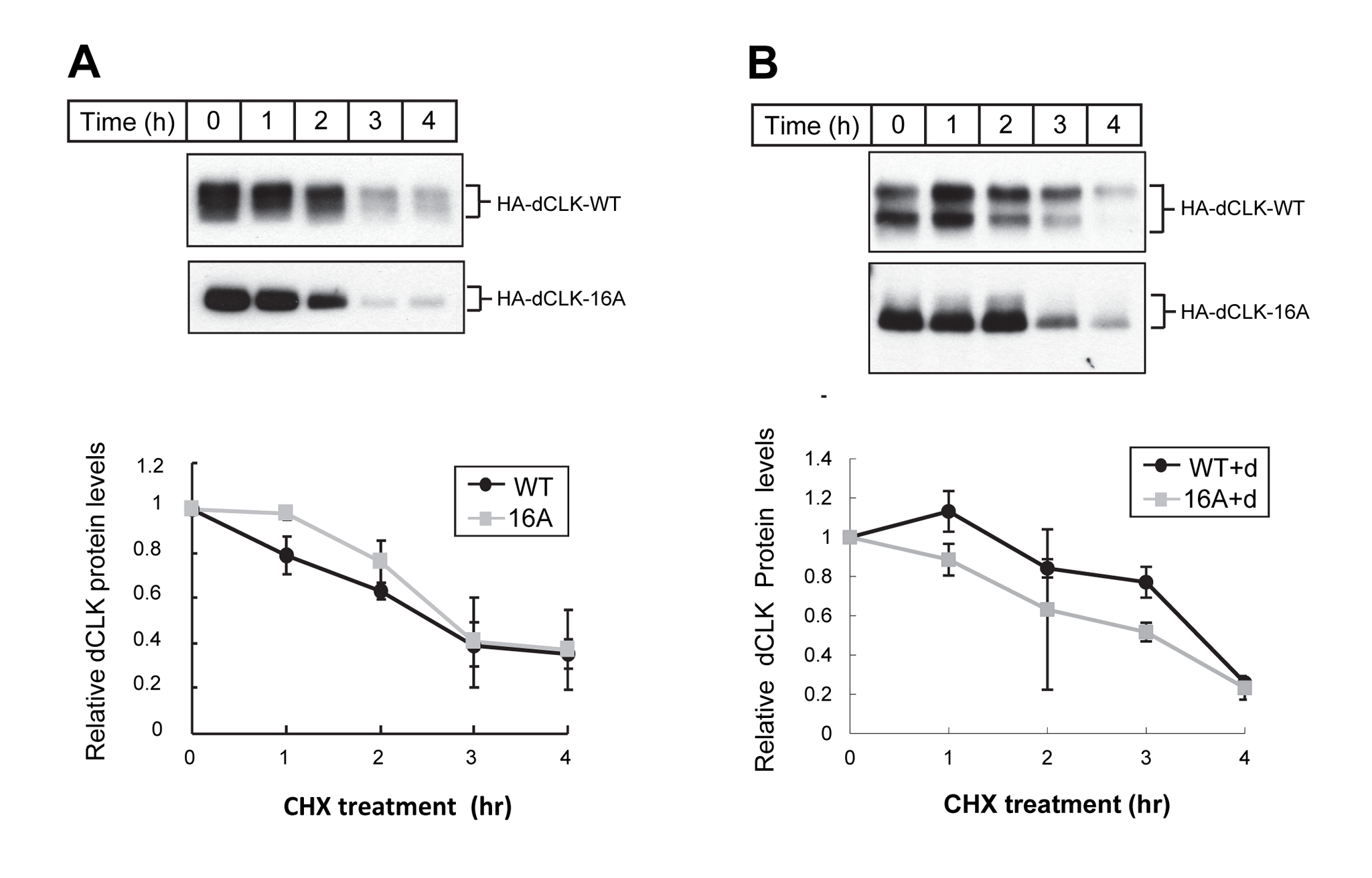

Supplement: Figure S2 — Stability of dCLK-WT and dCLK-16A protein in S2 cells. (A, B) S2 cells were transiently transfected with 300 ng of pMT-HA-dClk (WT) or pMT-HA-dClk-15A (15A) singly (A) or in combination with 600 ng of pMT-dbt-V5 (B). Expression of dCLK and DBT was induced 24 hr after transfection by adding 500 µM CuSO4 to the medium. 24 hrs post induction, 10 µg/ml of cycloheximide (CHX) was treated to inhibit translation. Cells were harvested at the indicated time points and protein extracts were subjected to immunoblotting. dCLK was visualized with anti-HA (3F10) antibody. Shown are the representative blots for each analysis and relative levels of dCLK proteins were determined by measuring band intensities of immunoblot using image J software. (TIF) [file pgen.1004545.s002.tif]

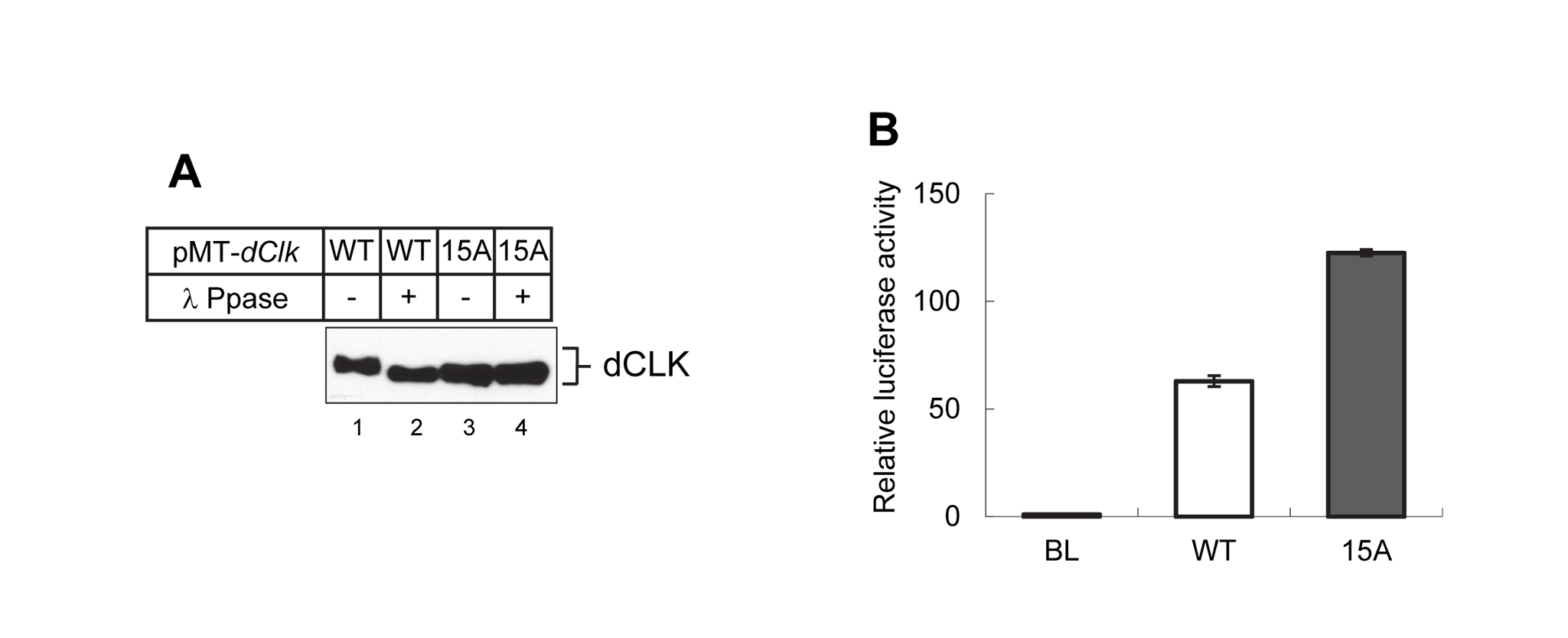

Supplement: Figure S3 — dCLK-15A manifests similar alterations as dCLK-16A compared to dCLK-WT in terms of stability and transcriptional activity in S2 cells. (A) S2 cells were transiently transfected with 500 ng of pMT-HA-dClk (WT) or pMT-HA-dClk-15A (15A). Expression of dCLK was induced 24 hr after transfection by adding 500 µM CuSO4 to the medium. Cells were harvested 24 hr after induction and protein extracts were first subjected to immunoprecipitation using anti-HA (12CA5) antibody and immune complexes were incubated in the absence (−) or presence (+) of λ phosphatase followed by immunoblotting. (B) Shown are the average values from three independent experiments for relative E box dependent luciferase activity in the absence (BL) or presence of pMT-dClk-V5 (WT) or pMT-dClk-15A (15A). (TIF) [file pgen.1004545.s003.tif]

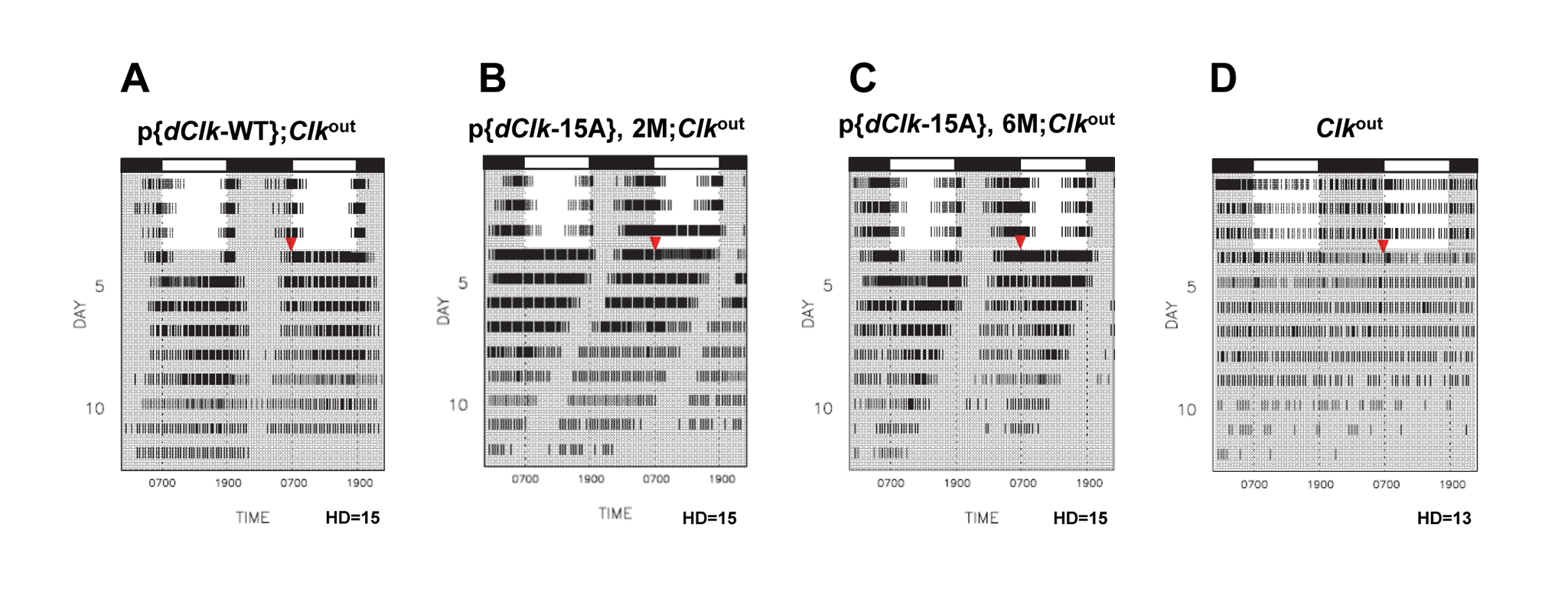

Supplement: Figure S4 — (A–D) Representative daily locomotor activity patterns of p{dClk-WT};Clk out and p{dClk-15A};Clk out flies in light/dark cycles. Adult flies of the indicated genotype (as indicated, top of panels) were entrained with 12 hr∶12 hr light∶dark cycles for 4 days followed by 8 days in DD. Black and white bar on top of each actogram indicates when lights were off and on, respectively. Red arrowhead indicates when DD starts. The vertical black bars on each row of the actogram depict the activity of the fly (measured in 30 min intervals). To better visualize rhythmic behavior, each day's worth of activity recordings was double plotted. HD, hash density of the actogram. (TIF) [file pgen.1004545.s004.tif]

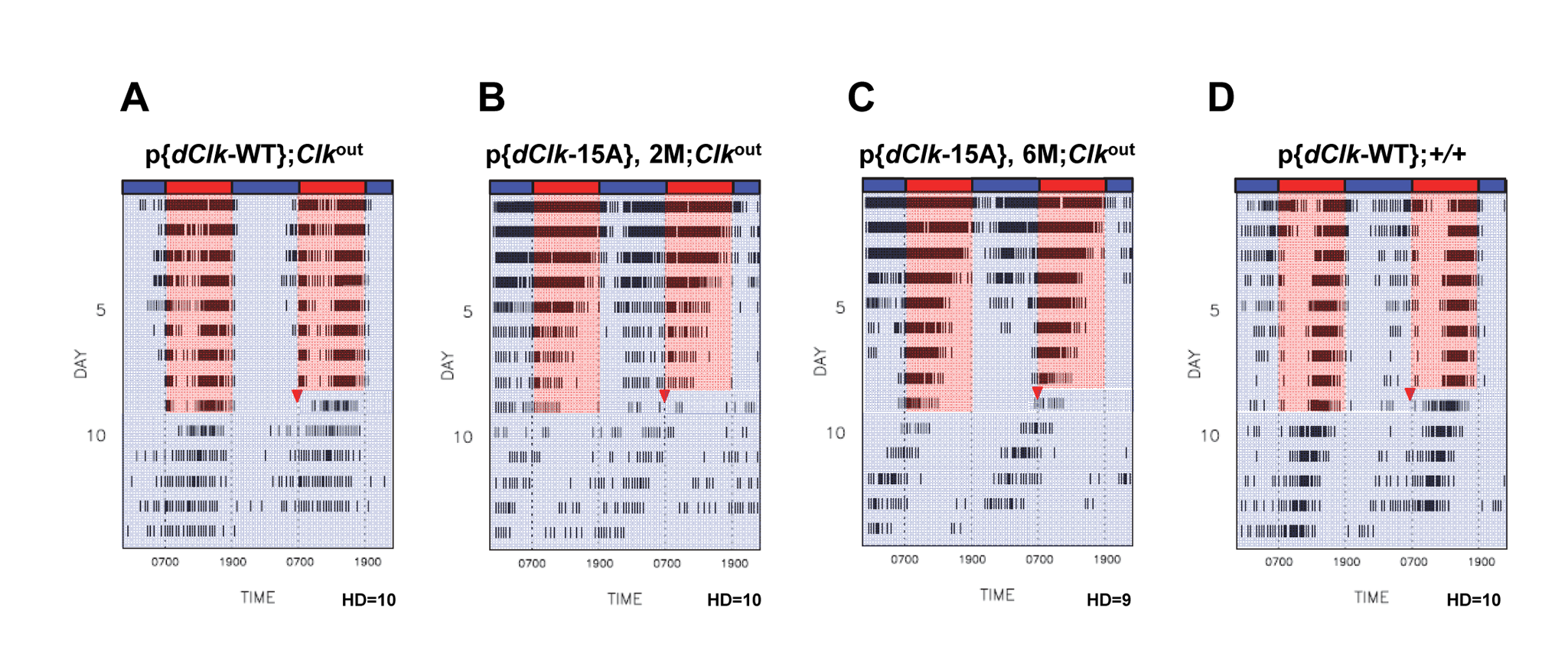

Supplement: Figure S5 — (A–D) Representative daily locomotor activities of p{dClk-WT};Clk out and p{dClk-15A};Clk out flies in temperature cycles in the absence of light. Adult male files for a given genotype (as indicated, top of panels) were entrained in 12 hr∶12 hr temperature cycles of 24°C∶29°C for 9 days and maintained at 24°C for 7 days in the absence light. The vertical black bars on each line of the actogram depict fly activity (measured in 30 min intervals). Each day's worth of activity recordings was double plotted to better visualize rhythmic behavior. Red horizontal bars and blue horizontal bars below each panel indicated thermo- or cryo-phases, respectively. The results clearly indicate that the offset in evening activity occurs progressively earlier in p{dClk-15A};Clk out flies even during TC. HD, hash density of the actogram. (TIF) [file pgen.1004545.s005.tif]

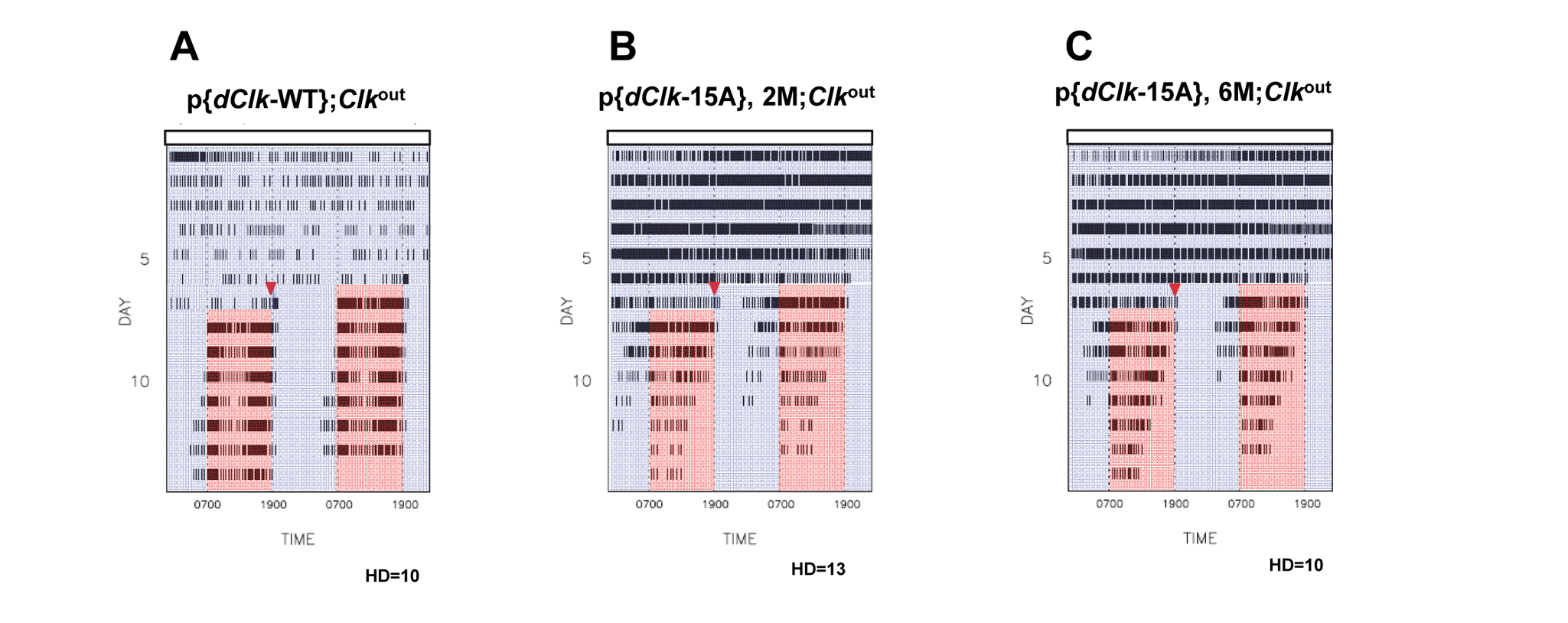

Supplement: Figure S6 — (A–C) Representative daily locomotor activities of p{dClk-WT};Clk out and p{dClk-15A};Clk out flies in temperature cycles after exposure to constant light. Adult male files for a given genotype (as indicated, top of panels) were exposed to constant light for 6 days and then entrained in 12 hr∶12 hr temperature cycles of 24°C∶29°C for 7 days in the absence light. The red arrowhead indicates when the lights were turned off. The vertical black bars on each line of the actogram depict fly activity (measured in 30 min intervals). Each day's worth of activity recordings was double plotted to better visualize rhythmic behavior. Flies became arrhythmic shortly after exposure to constant light. The results clearly indicate that the offset in evening activity occurs progressively earlier in p{dClk-15A};Clk out flies during TC. HD, hash density of the actogram. (TIF) [file pgen.1004545.s006.tif]

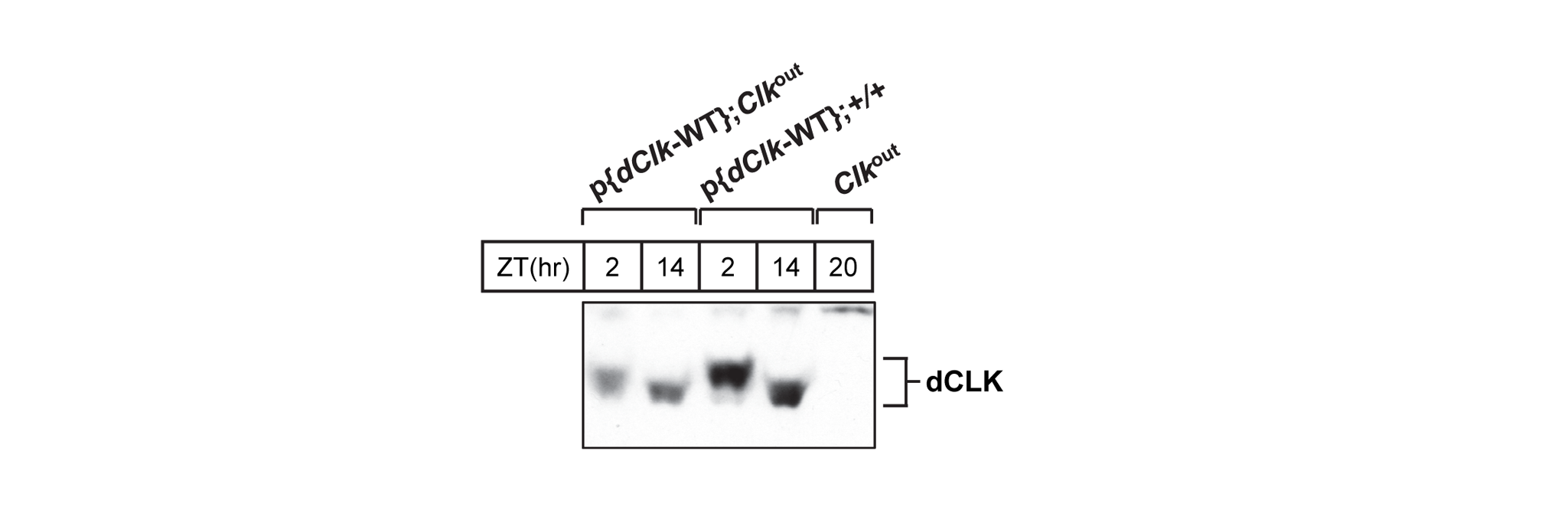

Supplement: Figure S7 — dCLK protein levels and phosphorylation in p{dClk-WT};Clk out and p{dClk-WT};+/+ flies. Adult flies of a given genotype (indicated at the top of panels) were collected at the indicated time in LD (ZT) and protein extracts analyzed by immunoblotting using the anti-dCLK antibody (gp208). Note that the levels of dCLK are higher in p{dClk-WT};+/+ flies. (TIF) [file pgen.1004545.s007.tif]
